# Collating the voice of people with autoimmune diseases: Methodology for the Third Phase of the COVAD Studies

**DOI:** 10.1007/s00296-024-05562-z

**Published:** 2024-04-12

**Authors:** Esha Kadam, Mahnoor Javaid, Parikshit Sen, Sreoshy Saha, Nelly Ziade, Jessica Day, Chris Wincup, Laura Andreoli, Ioannis Parodis, Ai Lyn Tan, Samuel Katsuyuki Shinjo, Dzifa Dey, Lorenzo Cavagna, Tulika Chatterjee, Johannes Knitza, Guochun Wang, Nicola Dalbeth, Tsvetelina Velikova, Simone Battista, Karen Cheng, Peter Boyd, Linda Kobert, Abraham Edgar Gracia-Ramos, Srijan Mittal, Ashima Makol, Carlos Enrique Toro Gutiérrez, Carlo V Caballero Uribe, Masataka Kuwana, Gerd-Rüdiger Burmester, Francis Guillemin, Elena Nikiphorou, Hector Chinoy, Vikas Aggarwal, Latika Gupta, the COVAD Study Group

**Affiliations:** 1 Seth Gordhandhas Sunderdas Medical College and King Edwards Memorial Hospital, Mumbai, Maharashtra India; 2 Medical College, Aga Khan University, Karachi, Pakistan; 3 Maulana Azad Medical College, 2-Bahadurshah Zafar Marg, New Delhi, Delhi 110002 India; 4 Mymensingh Medical College, Mymensingh, Bangladesh; 5 Rheumatology Department, Saint-Joseph University, Beirut, Lebanon; 6 Rheumatology Department, Hotel-Dieu de France Hospital, Beirut, Lebanon; 7 Department of Rheumatology, Royal Melbourne Hospital, Parkville, VIC 3050 Australia; 8 Walter and Eliza Hall Institute of Medical Research, Parkville, VIC 3052 Australia; 9 Department of Medical Biology, University of Melbourne, Parkville, VIC 3052 Australia; 10 Department of Rheumatology, King’s College Hospital, London, UK; 11 Rheumatology and Clinical Immunology Unit, ASST Spedali Civili and University of Brescia, 25123 Brescia, Italy; 12 Department of Clinical and Experimental Sciences, University of Brescia, 25123 Brescia, Italy; 13 Division of Rheumatology, Department of Medicine Solna, Karolinska Institutet and Karolinska University Hospital, Stockholm, Sweden; 14 Department of Rheumatology, Faculty of Medicine and Health, Örebro University, Örebro, Sweden; 15 NIHR Leeds Biomedical Research Centre, Leeds Teaching Hospitals Trust, Leeds, UK; 16 Leeds Institute of Rheumatic and Musculoskeletal Medicine, University of Leeds, Leeds, UK; 17 Division of Rheumatology, Faculdade de Medicina FMUSP, Universidade de São Paulo, São Paulo, SP Brazil; 18 Department of Medicine and Therapeutics, School of Medicine and Dentistry, University of Ghana, College of Health Sciences, Korle-Bu, Accra, Ghana; 19 Rheumatology Unit, Dipartimento di Medicine Interna e Terapia Medica, Università degli Studi di Pavia, Pavia, Lombardy Italy; 20 Department of Internal Medicine, University of Illinois College of Medicine at Peoria, Peoria, IL USA; 21 Medizinische Klinik 3, Rheumatologie Und Immunologie, Universitätsklinikum Erlangen, Friedrich-Alexander-Universität Erlangen-Nürnberg, Ulmenweg 18, 91054 Erlangen, Deutschland; 22 Department of Rheumatology, China-Japan Friendship Hospital, Yinghua East Road, Chaoyang District, Beijing, 100029 China; 23 Department of Medicine, University of Auckland, Auckland, New Zealand; 24 Medical Faculty, Sofia University, St. Kliment Ohridski, 1 Kozyak Str., 1407 Sofia, Bulgaria; 25 Department of Neurosciences, Rehabilitation, Ophthalmology, Genetics, Maternal and Child Health, Campus of Savona, University of Genova, Savona, Italy; 26 Patient Research Partner/Volunteer, Myositis Support and Understanding, Lincoln, USA; 27 Services Support Officer, Arthritis Ireland, Dublin, Ireland; 28 Chair, EULAR PARE Committee, Kilchberg, Switzerland; 29 Research and Communications Specialist, The Myositis Association, Lincoln, USA; 30 Department of Internal Medicine, National Medical Center “La Raza”, Instituto Mexicano del Seguro Social, General Hospital, Av. Jacaranda S/N, Col. La Raza, Del. Azcapotzalco, C.P. 02990 Mexico City, Mexico; 31 Division of Rheumatology, Mayo Clinic, Rochester, MN USA; 32 Reference Center for Osteoporosis, Rheumatology and Dermatology, Pontificia Universidad Javeriana Cali, Columbia, USA; 33 Department of Medicine, Hospital Universidad del Norte, Barranquilla, Atlantico Colombia; 34 Department of Allergy and Rheumatology, Nippon Medical School Graduate School of Medicine, 1-1-5 Sendagi, Bunkyo-Ku, Tokyo, 113-8602 Japan; 35 Department of Rheumatology and Clinical Immunology, Charité, University Medicine Berlin, Free University and Humboldt University Berlin, Berlin, Germany; 36 EA 4360 Apemac, Université de Lorraine, Nancy, France; 37 Inserm, CHRU Nancy, CIC-1433 Epidémiologie Clinique, Université de Lorraine, Nancy, France; 38 Centre for Rheumatic Diseases, King’s College London, London, UK; 39 Rheumatology Department, King’s College Hospital, London, UK; 40 Division of Musculoskeletal and Dermatological Sciences, Centre for Musculoskeletal Research, School of Biological Sciences, The University of Manchester, Manchester, UK; 41 National Institute for Health Research Manchester Biomedical Research Centre, Manchester University NHS Foundation Trust, The University of Manchester, Manchester, UK; 42 Department of Rheumatology, Salford Royal NHS Foundation Trust, Manchester Academic Health Science Centre, Salford, UK; 43 Department of Clinical Immunology and Rheumatology, Sanjay Gandhi Postgraduate Institute of Medical Sciences, Lucknow, India; 44 Department of Rheumatology, Royal Wolverhampton Hospitals NHS Trust, Wolverhampton, UK

**Keywords:** Autoimmune diseases, COVAD, Quality of life, Sociodemographic factors, Survey, Digital health

## Abstract

**Introduction:**

The growing recognition of holistic patient care highlights the various factors shaping the quality of life of individuals with autoimmune and rheumatic diseases (AIRDs). Beyond the traditional disease measures, there is an emerging acknowledgment of the less-explored aspects, including subjective well-being, social determinants of health, comorbidities, mental health, and medication adherence. Moreover, digital health services have empowered patients to engage actively in decision-making alongside clinicians. To explore these domains within the context of AIRDs, the “Collating the Voice of People with Autoimmune Diseases” COVAD survey was conceived, a successor of the previous two COVAD surveys. In this document, we present the study protocol in comprehensive detail.

**Methods:**

The COVAD-3 survey is a cross-sectional patient self-reported e-survey incorporating multiple widely accepted scales/scores to assess various aspects of patients’ lifestyles objectively. To ensure the survey's accuracy and usability across diverse regions, it will be translated into multiple languages and subjected to rigorous vetting and pilot testing. It will be distributed by collaborators via online platforms and data will be collected from patients with AIRDs, and healthy individuals over eight months. Data analysis will focus on outcome measures related to various social, demographic, economic, and psychological factors.

**Conclusion:**

With the increasing awareness to adopt a holistic treatment approach encompassing all avenues of life, the COVAD-3 survey aims to gain valuable insights into the impact of social, demographic, economic, and psychological determinants of health on the subjective well-being in patients with AIRDs, which will contribute to a better understanding of their overall health and well-being.

**Supplementary Information:**

The online version contains supplementary material available at 10.1007/s00296-024-05562-z.

## Introduction

Contemporary medical practice now recognizes the significance of clinical and biological factors and social, demographic, economic, and psychological factors in influencing disease activity and outcomes. Besides treatment based on an understanding of disease pathogenesis, social, psychological, and environmental factors need to be taken into account as part of holistic treatment. Consequently, a more comprehensive and holistic model for patient care, known as the biopsychosocial model [1], has emerged. This model considers all aspects of a patient's life that impact their quality of life, going beyond the traditional biomedical approach.

Although this model has been adopted for other severe diseases, such a model has not yet been widely embraced in the case of AIRDs. Therefore, further research in this patient population is essential to raise awareness and assist clinicians in incorporating similar holistic models into their treatment strategies. By doing so, healthcare professionals can better address the complex needs of patients with AIRDs and improve their overall well-being and disease management.

Social determinants of health (SDH) encompass how individuals are born, grow, work, and live, along with the broader economic, social, and political systems that influence their daily lives. These factors include access to healthcare, employment opportunities, socioeconomic status, medical security, and insurance coverage. AIRDs represent chronic and persistent conditions characterized by recurrent flares and the gradual accumulation of organ and tissue damage. These conditions are marked by significant pain [2], fatigue, restricted mobility, and limitations in daily activities, which can be exacerbated by challenging living conditions. The impact of SDH on health outcomes is particularly pronounced in patients with AIRDs [3]. Moreover, the post-COVID syndrome, defined as the emergence of new symptoms within three months of the initial COVID-19 infection that lasted two months [4], is predominantly characterized by fatigue [5] and, hence, may aggravate fatigue and pain from a pre-existing AIRD, further impairing the quality of life.

Poor quality of life in patients with AIRDs can translate into impaired subjective well-being (SWB) [6], defined as an individual's assessment of life satisfaction, happiness, and overall well-being. The SDH can potentially impact SWB described above and, hence, assume a pivotal role in current times with a shift to digital approaches and electronic patient-reported outcome measures (ePROM), wherein triage pathways tend to limit an understanding of the social determinants of poor health and put people at risk for incorrect assessment.

While genetic differences contribute to the variability in disease manifestation, social, demographic, economic, and psychological factors also significantly determine how the disease affects different individuals. For instance, studies have demonstrated that patients with rheumatoid arthritis (RA) from lower socioeconomic backgrounds experience worse disease activity, poorer physical and mental health, and a lower quality of life than those with higher socioeconomic status [7].

Additionally, sociodemographic factors in patients with AIRDs have been linked to higher pain levels, deteriorating mental health, and overall poorer quality of life [2, 8]. These findings underscore the importance of considering the broader context of a patient's life and socioeconomic situation when providing care and treatment for AIRDs. Addressing social determinants of health can lead to more effective and holistic management of these diseases and ultimately improve patients' well-being and health outcomes. Despite the new and improved treatments in AIRDs that potentially provide every patient with a higher chance of remission, patients’ response varies across a spectrum. This could be explained by several factors, including poor medication adherence, leading to poorer health outcomes and greater healthcare expenses [9–11]. Uncovering and catering to these factors would lead to greater uniformity and predictability in response to treatments.

In today’s technologically advancing world, several studies have underlined the impact of newly emerging technologies and artificial intelligence on healthcare [12], including the care of AIRDs [13]. Digital healthcare tools, including remote monitoring services, online applications, and electronic health records, have allowed patients to be equal partners to their clinicians in making important healthcare decisions. This has made it easier for the patient’s social, demographic, economic, and psychological conditions to be considered while building treatment plans. Extending these services to patients with AIRDs can improve their access to healthcare services; however, the usefulness of these tools in rare patient populations has not yet been gathered in detail. Moreover, the ease of use of these tools continues to be a hurdle for many patients.

The primary aim of this study is to move beyond the traditional focus on clinical and biological factors and delve into the influence of sociodemographic and technological determinants of health and, mental health and personal factors on SWB in patients with AIRDs. In alignment with this goal, we decided to rename the previous "COVID-19 Vaccination in Autoimmune Diseases" (COVAD) study, which primarily concentrated on disease-specific characteristics, to "Collating the Voice of Patients with AIRDs." This name change reflects our commitment to capturing a more comprehensive perspective.

In addition to the primary aim, the COVAD-3 study also aims to evaluate the impact of social, psychological, and environmental factors on the management of AIRDs. This will include disease activity, physical and social functionality, post-COVID syndrome, impact of digital healthcare tools, patient engagement in AIRDs management, outcomes, and quality of life.

## Methods

### Study group

The COVAD study is an international collaborative study that is aimed at embodying the voice of patients with AIRDs. The COVAD study achieved this by publishing extensively on patient-reported symptoms and outcomes to improve patient care. It was meticulously designed and reviewed by a multidisciplinary team of international experts, including doctors and researchers, forming the COVAD core team and steering Committee.

With 39,096 participants recruited to date, the COVAD database offers extensive geographical coverage, also involving under-represented regions. It utilizes validated tools such as the Patient-Reported Outcomes Measurement Information System (PROMIS) questionnaires [14]. The previous phases, COVAD-1 [15] and COVAD-2 [16] examined the short-term and long-term adverse effects of COVID-19 vaccinations in patients with AIRDs and disease-specific determinants of quality of life, including pain and fatigue in these patients.

COVAD-3 will be more comprehensive, aiming to assess the subjective well-being and how various socio-demographic factors impact the health outcomes and quality of life of people with AIRDs. The COVAD-3 survey will be built upon the same methodology and distribution strategy as COVAD-1 and COVAD-2 and will be extensively vetted and pilot-tested by the COVAD steering committee to ensure the survey's accuracy and reliability.

### Survey design

The survey questionnaire for the COVAD-3 study consists of a total of 125 questions and will approximately take 15 min to fill out the survey. The 125 questions are further subdivided into 13 specific subsections several of which are optional, covering various domains such as pre-existing AIRDs medication adherence, disease activity status, comorbidities, mental health, digital health, current health status, and quality of life, pain and dryness, social determinants of health, demographic information, pregnancy and lactation, sexual health and contraception, diet, exercise/physical activity.

The compulsory subsections are disease information comprising 15 questions; commodities comprising 7 questions; subjective well-being comprising 1 question; current health status and quality of life containing 13 questions; current disease activity status comprising 25 questions; medication adherence containing 8 questions; social determinants of health containing 15 questions; personal questions having 6 questions and mental health containing 3 questions. The optional subsections include pregnancy and lactation having 9 questions; sexual health and contraception having 3 questions; diet containing 15 questions; and exercise, physical activity, and digital health containing 3 questions. The last two questions of the survey will ask the respondents where they heard about the COVAD-3 survey and for their email addresses for follow-up regarding the survey.

The survey includes questions with single and multiple-answer selections, sliding-scale, dropdown options, and "other (please specify)" options for open-ended responses. Some questions are specifically for patients with AIRDs, while others apply to both health controls and AIRDs patients. However, the extensive use of logic functions will reduce the number of questions for the participants depending on the specific AIRD. The logic function has been used for 11 AIRD- Ankylosing Spondylitis, Gout, Sjögren’s Syndrome, Connective Tissue Diseases, Myositis, Overlap myositis, Scleroderma, Psoriatric Arthritis, Pseudogout, Systemic lupus erythematosus and Vasculitis. Refer to the survey attached for further details (Supplementary File).

Furthermore, the survey incorporates multiple widely accepted rating scales/scores (Table 1) to assess various aspects of patients' lifestyles objectively. Each of these scales/scores explores a unique theme among the different areas investigated in this survey.

**Table 1:** Summary information of the scales used in the COVAD-3 survey

Description	Scale	No. Of items	Domain/theme	Scaling
Number of comorbidities	Functional comorbidity index	18	1. Rheumatic/orthopedic comorbid 2. Cardiovascular comorbid 3. Respiratory comorbid 4. Neurological comorbid 5. Endocrine comorbid 6. Gastrointestinal comorbid 7. Psychiatric comorbid 8. Visual/auditory comorbid	Multiple responses
Subjective well-being	Satisfaction with life scale (SWLS)	5	N.A	7-point Likert scale (1–7)
Current health status	PROMIS GLOBAL-10	10	1. Physical health 2. Mental health 3. Social health	Semantic differential scale (1–5)
Fatigue	Visual analogue scale-Fatigue (VAS-F)	1	N.A	Semantic differential scale (0–10)
Pain	Visual analogue scale-Pain (VAS-P)	1	N.A	Semantic differential scale (0–10)
Primary Sjögren’s Syndrome Disease Activity	EULAR Sjögren’s Syndrome Patient Reported Index (ESSPRI)	3	1. Dryness 2. Limb pain 3. Fatigue	Semantic differential scale (0–10)
Physical function	PROMIS PF-4	4	N.A	Semantic differential scale (1–5)
Self-efficacy	Self-Efficacy For Managing Chronic Disease Scale	6	1. Symptom control 2. Role function 3. Emotional functioning 4. Communicating with clinicians	Semantic differential scale (1–10)
Disease activity	Patient Global Disease Activity Score	1	Effect of autoimmune/rheumatic disease on patient	Semantic differential scale (0–10)
Damage from disease	Patient Global Damage Assessment Score	1	Damage caused by autoimmune/rheumatic disease on the patient's body	Semantic differential scale (0–10)
Psoriatic arthritis disease activity	The EULAR Psoriatic Arthritis Impact of Disease: PsAID12 for clinical practice	12	1. Pain 2. Fatigue 3. Skin problems 4. Work and/or leisure activity 5. Functional capacity 6. Discomfort 7. Sleep disturbance 8. Coping 9. Anxiety, fear and uncertainty 10. Embarrassment and/or shame 11. Social participation 12. Depression	Semantic differential scale (0–10)
Ankylosing Spondylitis activity	Bath Ankylosing Spondylitis Disease Activity Index (BASDAI)	6	1. Fatigue/tiredness 2. Pain 3. Discomfort 4. Morning stiffness	Semantic differential scale (0–10)
Medication adherence	Morisky Medication Taking Adherence Scale (MMAS)	4	Adherence to anti-rheumatic medications	1. Yes/no question 2. Multiple choice questions
Trust in health insurance	Patient trust in a health insurer scale	5	N.A	5-point Likert scale (1–5)
Family functionality	Family APGAR scale	5	1. Adaptation 2. Partnership 3. Growth 4. Affection 5. Resolve	Semantic differential scale (0–3)
Job satisfaction	Short Index of Job Satisfaction	6	N.A	5-point Likert scale (1–5)
Loneliness	Loneliness Scale	3	1. Companionship 2. Isolation	Semantic differential scale (1–3)
Resilience	Brief Resilience Scale	6	N.A	5-point Likert scale (1–5)
Sexual Well-being	Short Sexual Well-being Scale (SSWBS)	5	1. Frequency 2. Sexual distress 3. Physical sexual satisfaction 4. Emotional sexual satisfaction 5. Sexuality in the social sphere	7-point Likert scale (1–7)
Diet	Mediterranean Diet Adherence Screener (MEDAS) questionnaire	14	N.A	1. Yes/no question 2. Multiple choice questions
Knowledge of, attitudes toward, and use of cancer- and health-related information	HINTS 6	17	1. Looking for health Information 2. Using the Internet to find information 3.Your healthcare 4. Telehealth 5. Medical records 6. Caregiving 7. Genetic testing 8. Overall Health 9. Environment and health 10. Social determinants of health 11. Health and nutrition 12. Physical activity and exercise 13. Tobacco products 14. Cancer screening and awareness 15. Beliefs about cancer 16. Cancer history 17. You and household	Multiple mixed scale type

### Current disease activity

The current disease activity status subsection will only be visible to respondents choosing they have ‘rheumatic disease’ in question 1 or ‘autoimmune disease’ in question 2. This subsection will not be visible to healthy respondents. The first questions the respondents are asked irrespective of their diagnosis are- who confirmed their AIRD diagnosis and in what year they were diagnosed. The Patient Global Disease Activity Assessment and Patient Global Damage Assessment scales (Table 1) have been widely used to determine the disease status in arthritis patients. These scales will be used to record the current disease activity based on the involvement of specific organs, including the joints and skin. In the Patient Global Disease Activity Assessment, responses will be recorded on a 10-point scale ranging from ‘no evidence of disease activity’ to ‘extremely active disease’ with a higher score reflecting greater disease activity. Similarly, answers in the Patient Global Damage Assessment scale are also marked along a 10-point scale ranging from ‘no evidence of damage’ to ‘extreme disease damage’ and a higher score indicating greater disease damage.

The Visual Analogue Scale (VAS) [17] (Table 1) is a single-item scale that will be used to determine the severity of pain in patients with the answers ranging from ‘no pain’ (scored 0) to ‘worst possible pain’ (scored 10). Fatigue will similarly be measured through the self reported visual analogue scale for fatigue (VAS-F) where ‘not at all fatigued’ will be scored 0 and ‘extremely fatigued’ will be scored 10. Higher score on VAS-F indicate greater perceived fatigue.

Logic functions have been widely used in this section to ask respondents about disease-specific activity. If the respondents select Ankylosing Spondylitis, questions on the activity of Ankylosing Spondylitis will be revealed. The activity of Ankylosing Spondylitis will be ascertained through the Bath Ankylosing Spondylitis Disease Activity Index (BASDAI) scale [18]. This is a 6-item long scale that will inquire about fatigue/tiredness, pain/swelling in joints, discomfort, and morning stiffness with answers between ‘none’ (scored 0) to ‘very severe’ (scored 10) for each item. An average score will be calculated to give a 0–10 BASDAI score, with a score of 4 or more suggesting a suboptimal control of the disease. Selection of Sjögren's syndrome will prompt the responders to fill out the European Alliance of Associations for Rheumatology (EULAR) Sjögren’s Syndrome Patient Reported Index (ESSPRI). The (ESSPRI) [19] is a 3-item long scale to determine the severity of disease in Sjögren’s syndrome patients specifically based on the presence or absence of dryness, fatigue, and limb pain. The responses for each item range from ‘no dryness/fatigue/pain’ to ‘maximum imaginable dryness/fatigue/pain’. A higher combined score would mean an increased severity of Sjögren’s syndrome.

On selecting either myositis, or overlap myositis, or scleroderma or vasculitis the logic function will prompt the respondents to answer what type of myositis/scleroderma/vasculitis they have. The myositis and overlap myositis logic chain will further ask the respondents about the presence of myositis antibodies. This similar logic chain is followed for Connective Tissue Disorder where respondents will have to answer about their Connective Tissue Disorder antibodies. In addition to the above questions, the overlap myositis logic chain will also ask about the additional disease they have in addition to myositis. Selection of Systemic lupus erythematosus (SLE) will lead to the respondent answering about the presence of lupus nephritis. Questions on the respondents being diagnosed with interstitial lung disease and Pulmonary Arterial Hypertension (PAH) will not be visible if Gout/Pseudogout/Calcium pyrophosphate deposition disease (CPPD) are chosen. They will instead be asked about the presence of tophi, the total number of gout attacks and consumption of medication specifically targeted towards Gout/Pseudogout and CPPD.

Selecting Psoriatic Arthritis will lead to the logic chain of questions ascertaining the activity of psoriatic arthritis. The activity of Psoriatic Arthritis will be ascertained through the 12-item EULAR Psoriatic Arthritis Impact of Disease: PsAID12 for clinical practice where the final PsAID12 is calculated in the range of 0–10 with 0 indicating the best status and 10 indicating the worst status [20]

### Medications and adherence

The Morisky Medication Adherence Scale (MMAS) [21] (Table 1) is a medication adherence scale with a validated use for rheumatic diseases. It is a 4-point scale that uses four items answered separately as yes/no and marked to determine adherence to anti-rheumatic medications. A higher score points to lower levels of medication adherence.

### Comorbidities

The Functional Comorbidity Index (Table 1) is an 18-item long scale to determine the presence of 18 comorbid conditions. It has been regarded as superior to its predecessor scales in determining the functionality of patients [22]. It is a multiple-response question with a yes/no answer to each question, so more questions marked as yes would mean a greater number of comorbid conditions.

### Current health status and quality of life

PROMIS scores (Table 1) are patient-centered questionnaires to assess their quality of life in general. The PROMIS GLOBAL-10 is a 10-item system to evaluate patients' physical, mental, and social health with answers ranging from ‘excellent’ to ‘poor’. The PROMIS PF-4 (4 items) assesses the ability to perform daily life activities with some answers ranging from ‘without any difficulty’ to ‘unable to do’. A higher score for each corresponds to better physical, mental, and social health. The Satisfaction With Life Scale (SWLS) [23] is a widely used, 5-item scale to assess SWB. Each item is rated between ‘strongly disagree’ and ‘strongly agree’, with the highest total score suggesting ‘extreme satisfaction’ and the lowest total score suggesting ‘extreme dissatisfaction’, respectively.

### Self-efficacy

The Self-Efficacy for Managing Chronic Disease Scale [24, 25] (Table 1) has been used for various chronic diseases, including chronic AIRDs. This scale consists of six items determining how confident the patient feels in managing their disease, with ten possible responses ranging from ‘not at all confident’ to ‘totally confident’. The total score for all six items will be determined, and a higher score will indicate greater self-efficacy and better ability to manage the disease.

### Mental health

The Loneliness scale [26] (Table 1) is a short questionnaire of 3 items to assess isolation and companionship in patients, with responses ranging from ‘hardly ever’ to ‘often’. A higher combined score indicates a greater degree of loneliness. Similarly, the Brief Resilience Scale (Table 1) assessed the patient’s ability to deal with difficult times through 6 items with responses varying from ‘strongly disagree’ to ‘strongly agree’. A higher score reflects greater resilience in managing stress.

### Social determinants of health

Several scales have been included to analyze the different social determinants of health separately, encompassing family and relationship dynamics. The Family APGAR scale [27] (Table 1) assesses five aspects of family functionality using five items with answers ranging from ‘hardly ever’ to ‘almost always’. A higher score on this scale indicates a highly functioning family.

The Short Index of Job Satisfaction (SIJS) [28] (Table 1) inquiries about patients’ satisfaction with their employment, with responses ranging from ‘strongly disagree’ to ‘strongly agree’. A higher score equates to greater job satisfaction. A few questions in the digital health section (about electronic wearable devices as well as health-related information on the internet) and in social determinants of health (regarding household income, marital status, and trust in the healthcare system) were taken from the HINTS 6 (2022) survey [29].

Finally, the Patient Trust in a healthcare insurer scale [30] (Table 1) determines how reliable the healthcare system is to the patients, particularly healthcare insurers. The scale has five items with responses ranging from ‘strongly disagree’ to ‘strongly agree’. A higher score means the patient has a greater trust in the healthcare system and the insurer.

### Sexual health

The Short Sexual Well-Being Scale (SSWBS) [31] is a 5-item questionnaire assessing sexual satisfaction, compliance, and distress. The answers range from ‘completely disagree’ (scored 1) to ‘completely agree’ (scored 7), with a higher score indicating a higher degree of sexual well-being.

### Diet

The optional diet section of the survey includes questions adapted from the Mediterranean Diet Adherence Screener (MEDAS) questionnaire [32]. This section aims to assess the participants' compliance with the Mediterranean diet, which is renowned for its extensive and scientifically proven health benefits [33].

The questionnaire begins with a yes/no item, while all subsequent questions require participants to select a number from the provided options. Each answer corresponds to a specific cutoff for scoring one point. The cumulative score from the answers determines the degree of adherence to the Mediterranean diet, with a higher score indicating greater adherence to the diet.

### Pilot testing and validation

The survey questions were reviewed by the steering committee members, who confirmed that it is representative of the aims and objectives of the study. The survey has underwent 40 rounds of testing by international experts which include rheumatologist, neurologist and internists. It also has undergone further testing by patients, the lay public, medical students and patient support groups to evaluate its face validity. All suggestions by the testers were taken into considerations and the survey was refined and retested until there were no changes to be made.

### Population selection

The study will include adult participants over 18, either healthy individuals or those diagnosed with autoimmune illnesses to allow comparative analysis. All individuals who consent electronically will be eligible to participate in the survey [34]. Convenience, snowball, and target sampling methods will be employed to recruit participants.

Upon accessing the survey link, participants will be presented with a cover letter containing detailed information about the survey. The cover letter will also request their informed consent for the study results to be published in a peer-reviewed journal. No incentives will be provided to the participants for survey completion, and they will remain anonymous unless they deliberately offer their contact details for follow-up purposes.

Using a combination of sampling methods and obtaining informed consent from the participants, the study aims to gather comprehensive data from individuals with diverse backgrounds and health statuses, contributing to a more robust and informative research outcome.

### Ethical considerations

Approval of the local Institutional Review Board (IRB) will be obtained as per local guidelines [34].

### Survey dissemination

COVAD collaborators will share the survey with their patients and approach patient support communities in the region to disseminate among their members. The survey will also be disseminated through social media platforms, allowing eight months for data collection.

### Statistical analysis

Data cleaning will be a crucial step in the study to ensure the accuracy and reliability of the collected data. It will involve the removal of duplicates, correcting structural errors, and handling outliers and missing data. Descriptive statistics will summarize the data, and inter-group comparisons will be conducted when appropriate. Individual study teams would participate in methods design, analysis, and writing following due approvals from the core team and steering committee.

For the "others (please specify)" category in open-ended responses, efforts will be made to reclassify the responses into existing categories. If a response does not fit into any existing category, a new one will be introduced.

Incomplete responses will be analysed and a cleaning strategy formulated for individual projects based on the extent and type of missing data to ensure the integrity of the results. Statistical software such as SPSS and R will be utilized for the subsequent analysis, allowing for data processing and analysis.

### Future analyses from the dataset

The anonymized dataset will be open to future analysis by the core team and the steering committee based on proposed hypotheses, research questions, and study designs approved by collaborators. The COVAD steering committee will vet and approve the proposals for scientific validity and feasibility, help translate the survey, and give intellectual input toward the survey design.

### Dissemination of results

Results will be disseminated in select peer-reviewed journals, on media, online, and at academic conferences. Respondents will be able to receive a plain language summary of the results upon request.

### Data sharing

Study leads will make study information, like study design, project administration, and publication preprints, available upon reasonable request.

### Project closure

After the study, online survey materials will be deactivated or removed. All data will remain securely stored centrally with the Project investigator for five years.

## Discussion

There is an increasing realization of the need to adopt a holistic management approach for people living with AIRDs, which makes it necessary to explore the social, demographic, economic, psychological, and emotional determinants of health. Such approaches have received traction as evidenced, e.g., by recent efforts to provide EULAR recommendations for the non-pharmacological management of such diseases [35]. The survey will explore all these avenues to ensure the validity of the dataset. In addition, to achieve validity, the questions will be kept short, straightforward, easy to comprehend, and non-leading, with all the possible responses included as answers. This large, geographically, and ethnically diverse dataset will make the survey applicable to a broad patient population worldwide, transcending borders and sociodemographic differences. Additionally, questions about patients’ access to and trust in the healthcare system, encompassing bothincludi public and private practices, will help identify areas of improvement and encourage discussion among policymakers to improve services, even for the small patient populations with rare diseases. Information on comorbid diseases and mental health conditions will underline how the co-occurence of diseases can lead to anxiety and depression, advocating for the adoption of the biopsychosocial model for patients with AIRDs. Inquiring about reasons behind poor medication adherence will allow clinicians to work around them during patient consultations [36].

Using validated assessment scales throughout the survey will ensure objectivity so that reliable results can be derived from the generated dataset. The wide array of items included in the survey will help patients and clinicians understand the impact of seemingly unrelated aspects of life on their illness, provide insight into the risks associated with the above confounding factors/disease parameters, and reduce variation in care. Our results will ultimately inform strategies to strengthen patient self-management, reduce anxiety surrounding health conditions, and reduce morbidity and mortality from adverse health outcomes. In addition, early identification of at-risk populations and implementation of multidisciplinary strategies may reduce patient-reported health outcome disparities [8].

Integrating digital technology within the healthcare system has helped transform it to make it more accessible and convenient. However, despite abundant research, less literature is available on the usefulness of these services to patients with rare AIRDs who have always had limited access to healthcare services compared to other patient populations. Hence, this study intends to explore this further by assessing the use of and access to digital tools and remote patient monitoring and its impact on health and disease outcomes in patients with AIRDs. This will inform new strategies to improve their quality, ensure more objective patient reporting, make them more user-friendly and accessible, and expand them, particularly for those with limited access to healthcare services.

Finally, this survey will aid in developing a multi-dimensional treatment approach that includes psychiatry, psychology, medicine, and rheumatology to devise well-informed guidelines to improve health outcomes and the quality of life for people living with AIRDs.

A major strength of this survey is that with the collaborators spanning 86 countries, a robust and inclusive database can be generated that accurately represents individuals of diverse genders, races, and ethnicities and is generalizable to a broad patient population. Additionally, the survey addresses a notable gap by including patients with rare AIRDs often overlooked in large-scale studies, thus making the survey results applicable to this underserved group. Finally, the survey will be disseminated to all institutions, regardless of their public or private status or specialization, to account for potential institutional variations.

A limitation of the survey is its length, which may discourage participants from completing the survey in full, leading to incomplete responses and data. These incomplete responses may not accurately represent individuals who did not submit a full response. Another limitation pertains to survey bias, which may introduce errors in recorded responses due to question misinterpretation or the influence of patients' personal beliefs and preferences.

## Conclusion

With the increasing awareness to adopt a holistic treatment approach encompassing all avenues of life, there is a need to adopt this approach for patients with AIRDs. The COVAD-3 survey will move beyond the traditional clinical and biological factors and collect data on social, demographic, economic, psychological, and technological determinants of health, and subjective well-being. This will capture a more comprehensive perspective of healthcare provision to these patients, allow patients to become equal partners to clinicians in healthcare decisions, and help develop multidisciplinary strategies to improve patient care.

## Supplementary Information

Below is the link to the electronic supplementary material.

Supplementary file 1 (DOCX 62 KB)

## Data Availability

The datasets generated and/or analyzed during the current study are not publicly available but are available from the corresponding author upon reasonable request.

## References

[1] Wade DT, Halligan PW (2017) The biopsychosocial model of illness: A model whose time has come. Clin Rehabil 31:995–1004 28730890 10.1177/0269215517709890

[2] Shinjo SK, Kim M, Hoff LS (2023) Pain in individuals with idiopathic inflammatory myopathies, other systemic autoimmune rheumatic diseases, and without rheumatic diseases: A report from the COVAD study. Int J Rheum Dis 26:727–739. 10.1111/1756-185X.14636 36872076 10.1111/1756-185X.14636

[3] Nikiphorou E, Alpizar-Rodriguez D, Gastelum-Strozzi A, Buch A, Peláez-Ballestas I (2021) Syndemics & syndemogenesis in COVID-19 and rheumatic and musculoskeletal diseases: old challenges, new era. Rheumatology (Oxford) 60:2040–2045. 10.1093/RHEUMATOLOGY/KEAA840 33496334 10.1093/rheumatology/keaa840 PMC7928641

[4] WHO (2022) Post COVID-19 condition (Long COVID). https://www.who.int/europe/news-room/fact-sheets/item/post-covid-19-condition. Accessed July 10, 2023

[5] Alghamdi SA, Alfares MA, Alsulami RA (2022) Post-COVID-19 Syndrome: Incidence, Risk Factor, and the Most Common Persisting Symptoms. Cureus. 10.7759/CUREUS.32058 36600841 10.7759/cureus.32058 PMC9802640

[6] Armadans-Tremolosa I, Selva-O’Callaghan A, Visauta-Vinacua B, Guilera G, Pinal-Fernández I, Vilardell-Tarrés M (2014) Health-related quality of life and well-being in adults with idiopathic inflammatory myopathy. Clin Rheumatol 33:1119–1125. 10.1007/S10067-014-2685-0 24894104 10.1007/s10067-014-2685-0

[7] Jacobi CE, Mol GD, Boshuizen HC, Rupp I, Dinant HJ, Van Den Bos GA (2003) Impact of socioeconomic status on the course of rheumatoid arthritis and on related use of health care services. Arthritis Rheum 49:567–573. 10.1002/ART.11200 12910565 10.1002/art.11200

[8] Kumaradev S, Roux C, Sellam J, Perrot S, Pham T, Dugravot A, Molto A (2022) Socio-demographic determinants in the evolution of pain in inflammatory rheumatic diseases: results from ESPOIR and DESIR cohorts. Rheumatology (Oxford) 61:1496–1509. 10.1093/RHEUMATOLOGY/KEAB562 34270700 10.1093/rheumatology/keab562 PMC8996788

[9] van Mierlo T, Fournier R, Ingham M (2015) Targeting Medication Non-Adherence Behavior in Selected Autoimmune Diseases: A Systematic Approach to Digital Health Program Development. PLoS ONE. 10.1371/journal.pone.0129364 26107637 10.1371/journal.pone.0129364 PMC4481109

[10] De Vera MA, Mailman J, Galo JS (2014) Economics of non-adherence to biologic therapies in rheumatoid arthritis topical collection on health economics and quality of life. Curr Rheumatol Rep 16:1–9. 10.1007/S11926-014-0460-5/TABLES/1 10.1007/s11926-014-0460-5 25227187

[11] Vangeli E, Bakhshi S, Baker A, Fisher A, Bucknor D, Mrowietz U, Ostor AJ, Peyrin-Biroulet L, Lacerda AP, Weinman J (2015) A Systematic Review of Factors Associated with Non-Adherence to Treatment for Immune-Mediated Inflammatory Diseases. Adv Ther 32(11):983–1028. 10.1007/s12325-015-0256-7 26547912 10.1007/s12325-015-0256-7 PMC4662720

[12] Stoumpos AI, Kitsios F, Talias MA (2023) Digital Transformation in Healthcare: Technology Acceptance and Its Applications. Int J Environ Res Public Health. 10.3390/ijerph20043407 36834105 10.3390/ijerph20043407 PMC9963556

[13] Stafford IS, Kellermann M, Mossotto E, Beattie RM, MacArthur BD, Ennis S (2020) A systematic review of the applications of artificial intelligence and machine learning in autoimmune diseases. NPJ Digit Med 3:30. 10.1038/s41746-020-0229-3 32195365 10.1038/s41746-020-0229-3 PMC7062883

[14] Health measures (nd) PROMIS https://www.healthmeasures.net/explore-measurement-systems/promis Accessed September 5, 2023

[15] Sen P, Gupta L, Lilleker JB (2022) COVID-19 vaccination in autoimmune disease (COVAD) survey protocol. Rheumatol Int 42:23–29. 10.1007/S00296-021-05046-4 34779868 10.1007/s00296-021-05046-4 PMC8591970

[16] Sen P, Gupta L, Lilleker JB, Aggarwal V, Kardes S, Milchert M, Gheita T, Salim B, Velikova T, Gracia-Ramos AE, Parodis I, O’Callaghan AS, Nikiphorou E, Tan AL, Cavagna L, Saavedra MA, Shinjo SK, Ziade N, Knitza J, Kuwana M, Cagnotto G, Nune A, Distler O, Chinoy H, Aggarwal V, Aggarwal R (2022) COVID-19 vaccination in autoimmune disease (COVAD) survey protocol. Rheumatol Int 42(1):23–29. 10.1007/s00296-021-05046-4 34779868 10.1007/s00296-021-05046-4 PMC8591970

[17] (2000) Glossary. Spine 25:3200–3202

[18] Garrett S, Jenkinson T, Kennedy LG, Whitelock H, Gaisford P, Calin A (1994) A new approach to defining disease status in ankylosing spondylitis: the Bath Ankylosing Spondylitis Disease Activity Index. J Rheumatol 21:2286–2291 7699630

[19] Seror R, Ravaud P, Mariette X, Bootsma H, Theander E, Hansen A, Ramos-Casals M, Dorner T, Bombardieri S, Hachulla E, Brun JG, Kruize AA, Praprotnik S, Tomsic M, Gottenberg JE, Devauchelle V, Devita S, Vollenweider C, Mandl T, Tzioufas A, Carsons S, Saraux A, Sutcliffe N, Vitali C, Bowman SJ (2011) EULAR Sjogren’s Syndrome Patient Reported Index (ESSPRI): development of a consensus patient index for primary Sjogren’s syndrome. Ann Rheum Dis 70(6):968–972. 10.1136/ard.2010.143743 21345815 10.1136/ard.2010.143743

[20] Gossec L, de Wit M, Kiltz U, Braun J, Kalyoncu U, Scrivo R, Maccarone M, Carton L, Otsa K, Sooaar I, Heiberg T, Bertheussen H, Canete JD, Sanchez Lombarte A, Balanescu A, Dinte A, de Vlam K, Smolen JS, Stamm T, Niedermayer D, Bekes G, Veale D, Helliwell P, Parkinson A, Luger T, Kvien TK, Taskforce EP (2014) A patient-derived and patient-reported outcome measure for assessing psoriatic arthritis: elaboration and preliminary validation of the Psoriatic Arthritis Impact of Disease (PsAID) questionnaire, a 13-country EULAR initiative. Ann Rheum Dis 73(6):1012–1019 24790067 10.1136/annrheumdis-2014-205207

[21] The Morisky Medication Adherence Scale: An Overview, (n.d.). https://www.pillsy.com/articles/the-morisky-medication-adherence-scale-definition-alternatives-and-overview (accessed January 1, 2024)

[22] Groll DL, To T, Bombardier C, Wright JG (2005) The development of a comorbidity index with physical function as the outcome. J Clin Epidemiol 58:595–602. 10.1016/j.jclinepi.2004.10.018 15878473 10.1016/j.jclinepi.2004.10.018

[23] Diener E, Emmons RA, Larsen RJ, Griffin S (1985) The Satisfaction With Life Scale. J Pers Assess 49:71–75. 10.1207/s15327752jpa4901_13 16367493 10.1207/s15327752jpa4901_13

[24] Lorig KR, Sobel DS, Ritter PL, Laurent D, Hobbs M (2001) Effect of a self-management program on patients with chronic disease. Eff Clin Pract 4(6):256–262 11769298

[25] Ritter PL, Lorig K (2014) The English and Spanish Self-Efficacy to Manage Chronic Disease Scale measures were validated using multiple studies. J Clin Epidemiol 67:1265–1273. 10.1016/j.jclinepi.2014.06.009 25091546 10.1016/j.jclinepi.2014.06.009

[26] Hughes ME, Waite LJ, Hawkley LC, Cacioppo JT (2004) A Short Scale for Measuring Loneliness in Large Surveys: Results From Two Population-Based Studies. Res Aging 26(6):655–672. 10.1177/0164027504268574 18504506 10.1177/0164027504268574 PMC2394670

[27] Smilkstein G (1978) The family APGAR: a proposal for a family function test and its use by physicians. J Fam Pract 6(6):1231–1239 660126

[28] Sinval J, Maroco J (2020) Short Index of Job Satisfaction: Validity evidence from Portugal and Brazil. PLoS ONE 15(4):e0231474. 10.1371/journal.pone.0231474 32287284 10.1371/journal.pone.0231474 PMC7156096

[29] (nd) HINTS. Health Information National Trends Survey. https://hints.cancer.gov/ Accessed September 17, 2023.

[30] Dugan E, Trachtenberg F, Hall MA (2005) Development of abbreviated measures to assess patient trust in a physician, a health insurer, and the medical profession. BMC Health Serv Res 5:64. 10.1186/1472-6963-5-64 16202125 10.1186/1472-6963-5-64 PMC1262715

[31] Gerymski R (2021) Short sexual well-being scale - A cross-sectional validation among transgender and cisgender people. Heal Psychol Rep 9:276–287. 10.5114/HPR.2021.102349 10.5114/hpr.2021.102349 PMC10694696 38084233

[32] Garcia-Conesa MT, Philippou E, Pafilas C, Massaro M, Quarta S, Andrade V, Jorge R, Chervenkov M, Ivanova T, Dimitrova D, Maksimova V, Smilkov K, Ackova DG, Miloseva L, Ruskovska T, Deligiannidou GE, Kontogiorgis CA, Pinto P (2020) Exploring the Validity of the 14-Item Mediterranean Diet Adherence Screener (MEDAS): A Cross-National Study in Seven European Countries around the Mediterranean Region. Nutrients. 10.3390/nu12102960 32992649 10.3390/nu12102960 PMC7601687

[33] Parodis I, Gomez A, Tsoi A, Chow JW, Pezzella D, Girard C, Stamm TA, Bostrom C (2023) Systematic literature review informing the EULAR recommendations for the non-pharmacological management of systemic lupus erythematosus and systemic sclerosis. RMD Open. 10.1136/rmdopen-2023-003297 37532469 10.1136/rmdopen-2023-003297 PMC10401222

[34] (n d) ICMR Ethical Guidelines. ICMR. https://ethics.ncdirindia.org/icmr_ethical_guidelines.aspx Accessed August 4, 2023

[35] Parodis I, Girard-Guyonvarc’h C, Arnaud L, Distler O, Domjan A, Van den Ende CHM, Fligelstone K, Kocher A, Larosa M, Lau M, Mitropoulos A, Ndosi M, Poole JL, Redmond A, Ritschl V, Alexanderson H, Sjoberg Y, von Perner G, Uhlig T, Varju C, Vriezekolk JE, Welin E, Westhovens R, Stamm TA, Bostrom C (2023) EULAR recommendations for the non-pharmacological management of systemic lupus erythematosus and systemic sclerosis. Ann Rheum Dis. 10.1136/ard-2023-224416 10.1136/ard-2023-224416 37433575

[36] Emamikia S, Gentline C, Enman Y, Parodis I (2022) How Can We Enhance Adherence to Medications in Patients with Systemic Lupus Erythematosus? Results from a Qualitative Study. J Clin Med. 10.3390/JCM11071857 35407466 10.3390/jcm11071857 PMC8999748

