# Correction to: Collating the voice of people with autoimmune diseases: Methodology for the third phase of the COVAD studies

**DOI:** 10.1007/s00296-024-05692-4

**Published:** 2024-10-14

**Authors:** Esha Kadam, Mahnoor Javaid, Parikshit Sen, Sreoshy Saha, Nelly Ziade, Jessica Day, Chris Wincup, Laura Andreoli, Ioannis Parodis, Ai Lyn Tan, Samuel Katsuyuki Shinjo, Dzifa Dey, Lorenzo Cavagna, Tulika Chatterjee, Johannes Knitza, Guochun Wang, Nicola Dalbeth, Tsvetelina Velikova, Simone Battista, Karen Cheng, Peter Boyd, Linda Kobert, Abraham Edgar Gracia-Ramos, Srijan Mittal, Ashima Makol, Carlos Enrique Toro Gutiérrez, Carlo V. Caballero-Uribe, Masataka Kuwana, Gerd-Rüdiger Burmester, Francis Guillemin, Elena Nikiphorou, Hector Chinoy, Vikas Agarwal, Latika Gupta

**Affiliations:** 1 Seth Gordhandhas Sunderdas Medical College and King Edwards Memorial Hospital, Mumbai, Maharashtra India; 2 Medical College, Aga Khan University, Karachi, Pakistan; 3 Maulana Azad Medical College, 2-Bahadurshah Zafar Marg, New Delhi, Delhi 110002 India; 4 Mymensingh Medical College, Mymensingh, Bangladesh; 5 Rheumatology Department, Saint-Joseph University, Beirut, Lebanon; 6 Rheumatology Department, Hotel-Dieu de France Hospital, Beirut, Lebanon; 7 Department of Rheumatology, Royal Melbourne Hospital, Parkville, VIC 3050 Australia; 8 Eliza Hall Institute of Medical Research, Parkville, VIC 3052 Australia; 9 Department of Medical Biology, University of Melbourne, Parkville, VIC 3052 Australia; 10 Department of Rheumatology, King’s College Hospital, London, UK; 11 Rheumatology and Clinical Immunology Unit, ASST Spedali, Civili and University of Brescia, 25123 Brescia, Italy; 12 Department of Clinical and Experimental Sciences, University of Brescia, 25123 Brescia, Italy; 13 Division of Rheumatology, Department of Medicine Solna, Karolinska Institutet and Karolinska University Hospital, Stockholm, Sweden; 14 Department of Rheumatology, Faculty of Medicine and Health, Örebro University, Örebro, Sweden; 15 NIHR Leeds Biomedical Research Centre, Leeds Teaching Hospitals Trust, Leeds, UK; 16 Leeds Institute of Rheumatic and Musculoskeletal Medicine, University of Leeds, Leeds, UK; 17 Division of Rheumatology, Faculdade de Medicina FMUSP, Universidade de São Paulo, São Paulo, SP Brazil; 18 Department of Medicine and Therapeutics, School of Medicine and Dentistry, University of Ghana, College of Health Sciences, Korle-Bu, Accra, Ghana; 19 Rheumatology Unit, Dipartimento Di Medicine Interna E Terapia Medica, Università Degli Studi Di Pavia, Pavia, Lombardy Italy; 20 Department of Internal Medicine, University of Illinois College of Medicine at Peoria, Peoria, IL USA; 21 Medizinische Klinik 3, Rheumatologie Und Immunologie, Universitätsklinikum Erlangen, Friedrich-Alexander-Universität Erlangen-Nürnberg, Ulmenweg 18, 91054 Erlangen, Deutschland; 22 Department of Rheumatology, China-Japan Friendship Hospital, Yinghua East Road, Chaoyang District, Beijing, 100029 China; 23 Department of Medicine, University of Auckland, Auckland, New Zealand; 24 Medical Faculty, Sofia University, St. Kliment Ohridski, 1 Kozyak Str., 1407 Sofia, Bulgaria; 25 Department of Neurosciences, Rehabilitation, Ophthalmology, Genetics, Maternal and Child Health, Campus of Savona, University of Genova, Savona, Italy; 26 Associate Board Member, International Myositis Society, Göttingen, Germany; 27 Patient Advisor, MIHRA Foundation, New Orleans, USA; 28 Patient Research Partner, Myositis Support and Understanding, Lincoln, USA; 29 Services Support Ofcer, Arthritis Ireland, Dublin, Ireland; 30 EULAR PARE Committee, Kilchberg, Switzerland; 31 Research and Communications Specialist, The Myositis Association, Columbia, MD USA; 32 Department of Internal Medicine, National Medical Center “La Raza”, Instituto Mexicano del Seguro Social, General Hospital, Av. Jacaranda S/N, Col. La Raza, Del. Azcapotzalco, C.P. 02990 Mexico City, Mexico; 33 Division of Rheumatology, Mayo Clinic, Rochester, MN USA; 34 Reference Center for Osteoporosis, Rheumatology and Dermatology, Pontifcia Universidad Javeriana Cali, Cali, Colombia; 35 Department of Medicine, Hospital Universidad del Norte, Barranquilla, Atlantico Colombia; 36 Department of Allergy and Rheumatology, Nippon Medical School Graduate School of Medicine, 1-1-5 Sendagi, Bunkyo-Ku, Tokyo, 113-8602 Japan; 37 Department of Rheumatology and Clinical Immunology, Charité, University Medicine Berlin, Free University and Humboldt University Berlin, Berlin, Germany; 38 EA 4360 Apemac, Université de Lorraine, Nancy, France; 39 Inserm, CHRU Nancy, CIC-1433 Epidémiologie Clinique, Université de Lorraine, Nancy, France; 40 Centre for Rheumatic Diseases, King’s College London, London, UK; 41 Rheumatology Department, King’s College Hospital, London, UK; 42 Division of Musculoskeletal and Dermatological Sciences, Centre for Musculoskeletal Research, School of Biological Sciences, The University of Manchester, Manchester, UK; 43 National Institute for Health Research Manchester Biomedical Research Centre, Manchester University NHS Foundation Trust, The University of Manchester, Manchester, UK; 44 Department of Rheumatology, Salford Royal NHS Foundation Trust, Manchester Academic Health Science Centre, Salford, UK; 45 Department of Clinical Immunology and Rheumatology, Sanjay Gandhi Postgraduate Institute of Medical Sciences, Lucknow, India; 46 Department of Rheumatology, Royal Wolverhampton Hospitals NHS Trust, Wolverhampton, UK

**Keywords:** Autoimmune diseases, Quality of life, Sociodemographic factors, Survey, Correction, Digital health

**Correction** **:** **Rheumatology International (2024) 44:1233–1244** 10.1007/s00296-024-05562-z 

In the article by Kadam et al., the following corrections have been made:

1. The name of one co-author has been corrected to "Carlo V. Caballero-Uribe", which was previously incorrectly written as "Carlo V Caballero Uribe".
2. The affiliations of three co-authors have been corrected as following:
3. Karen Cheng—Associate Board Member, International Myositis Society, Göttingen, Germany; Patient Advisor, MIHRA Foundation, New Orleans, USA; Patient Research Partner, Myositis Support and Understanding, Lincoln, USA
4. Linda Kobert—Research and Communications Specialist, The Myositis Association, Columbia, MD, USA
5. Carlos Enrique Toro-Gutiérrez—Reference Center for Osteoporosis, Rheumatology and Dermatology, Pontificia Universidad Javeriana Cali, Colombia

The "Survey Design" section have been updated:

- "The survey questionnaire for the COVAD-3 study consists of a total of 141 questions and will approximately take 15 min to fill out the survey. The 141 questions are further subdivided into 13 specific subsections, several of which are optional….."
- Reference number 16 has been updated.
- Four new references have been added the "Current Disease Activity" section:

"The Patient Global Disease Activity Assessment and Patient Global Damage Assessment scales (Table 1) have been widely used to determine the disease status in arthritis patients." [37].

**Table 1:** Scales included in the COVAD-3 survey

Description	Scale	No. of items	Domain/Theme	Scaling
Number of comorbidities	Functional Comorbidity Index [22]	18	Rheumatic/orthopedic comorbid, Cardiovascular comorbid, Respiratory comorbid, Neurological comorbid, Endocrine comorbid, Gastrointestinal comorbid, Psychiatric comorbid, Visual/auditory comorbid	Multiple responses
Subjective well-being	Satisfaction With Life Scale (SWLS) [23]	5	N.A	7-point Likert scale (1–7)
Current health status	PROMIS GLOBAL Health-10 [14]	10	Physical health, Mental health, Social health	Semantic differential scale (1–5)
Fatigue	Visual Analogue Scale (VAS) [39]	1	N.A	Semantic differential scale (0–10)
Pain	Visual Analogue Scale (VAS) [42]	1	N.A	Semantic differential scale (0–10)
Physical function	PROMIS PF-4a [14]	4	N.A	Semantic differential scale (1–5)
Self-Efficacy	Self-Efficacy for Managing Chronic Disease Scale [24,25]	6	Symptom control, Role function, Emotional functioning, Communicating with clinicians	Semantic differential scale (1–10)
Disease activity	Patient Global Disease Activity scale [37,38]	1	Effect of autoimmune/rheumatic disease on patient	Semantic differential scale (0–10)
Damage Assessment	Patient Global Damage Assessment Scale [37,38]	1	Damage caused by autoimmune/rheumatic disease on the patient's body	Semantic differential scale (0–10
Trust in health insurance	Patient trust in a health insurer scale [30]	5	N.A	5-point Likert scale (1–5)
Family functionality	Family APGAR scale [27]	5	Adaptation, partnership, growth, affection, and resolve	Semantic differential scale (0–3)
Job satisfaction	Short Index of Job Satisfaction [28]	6	N.A	5-point Likert scale (1–5)
Loneliness	UCLA 3-Item Loneliness Scale [26]	3	Companionship and isolation	Semantic differential scale (1–3)
Resilience	Brief Resilience Scale [40]	6	N.A	5-point Likert scale (1–5)
Sexual Well-being	Short Sexual Well-being Scale (SSWBS) [31]	5	Frequency, sexual distress, physical sexual satisfaction, emotional sexual satisfaction, and sexuality in the social sphere	7-point Likert scale (1–7)
Diet	Mediterranean Diet Adherence Screener (MEDAS) questionnaire [32]	14	N.A	1. Yes/no question 2. Multiple choice questions
Knowledge of, attitudes toward, and use of health-related information	Health Information National Trends Survey (HINTS 6) [29]	14	Looking for health information, using the Internet to find information, your healthcare, telehealth, medical records, caregiving, genetic testing, overall health, environment and health, social determinants of health, health and nutrition, physical activity and exercise, tobacco products, and you and your household	Multiple mixed scale type
Ankylosing Spondylitis activity	Bath Ankylosing Spondylitis Disease Activity Index (BASDAI) [18]	6	Fatigue/tiredness, pain, discomfort and morning stiffness	Semantic differential scale (0–10)
Psoriatic arthritis disease activity	The EULAR Psoriatic Arthritis Impact of Disease: PsAID12 for Clinical Practice [20]	12	Pain, fatigue, skin problems, work and/or leisure activity, functional capacity, discomfort, sleep disturbance, coping, anxiety, fear and uncertainty, embarrassment and/or shame, social participation, and depression	Semantic differential scale (0–10)
Sjogren syndrome disease activity	EULAR Sjögren’s Syndrome Patient Reported Index (ESSPRI) [19]	3	Dryness, limb pain, and fatigue	Semantic differential scale (0–10)
Functional scale for myasthenia gravis	Myasthenia Gravis Activities of Daily Living [41]	8	Talking, chewing, swallowing, breathing, brushing teeth or hair, arising from chair, double vision, and eyelid drooping	Multiple mixed scale type

"These scales will be used to record the current disease activity based on the involvement of specific organs, including the joints and skin." [38].

"Fatigue will similarly be measured through the self-reported visual analogue scale for fatigue (VAS-F), where 'not at all fatigued' will be scored 0 and 'extremely fatigued' will be scored 10." [39].

The Visual Analogue Scale (VAS) (Table 1) is a single-item scale that will be used to determine the severity of pain in patients with the answers ranging from ‘no pain’ (scored 0) to ‘worst possible pain’ (scored 10). [42].

4. The "Methods" section has been updated:
• In "Medication and Adherence" subsection, this sentence has been added for clarification: "A question aims to assess how well the respondents follow their prescribed treatment plan, as medication adherence can affect outcomes in autoimmune diseases." Previous sentences have been discarded.
• Reference 21 has been omitted.
5. One new reference has been added in the "Mental Health" section to clarify the resilience scale:

"A higher score reflects greater resilience in managing stress." [40].

6. A revised version of Table 1 and the COVAD 3 survey (Supplementary material) are now available.
7. The declaration of one of the co-author has been updated:

“KC is a patient living with myositis and serves on the board of International Myositis Society, patient advisory group of the MIHRA foundation and EU patient advocacy for MSU”.

The identified errors do not invalidate the study's overall methodology. The authors apologize for any inconvenience caused and appreciate the opportunity to correct the record.

**New References to be updated.**

16. Fazal, Z.Z., Sen, P., Joshi, M. et al. COVAD survey 2 long-term outcomes: unmet need and protocol. Rheumatol Int 42, 2151–2158 (2022). 10.1007/s00296-022-05157-6.

37. Nikiphorou E, Radner H, Chatzidionysiou K, Desthieux C, Zabalan C, van Eijk-Hustings Y, Dixon WG, Hyrich KL, Askling J, Gossec L. Patient global assessment in measuring disease activity in rheumatoid arthritis: a review of the literature. Arthritis Res Ther. 2016 Oct 28;18(1):251. 10.1186/s13075-016-1151-6. PMID: 27,793,211; PMCID: PMC5086038.

38. Rider LG, Feldman BM, Perez MD, Rennebohm RM, Lindsley CB, Zemel LS, Wallace CA, Ballinger SH, Bowyer SL, Reed AM, Passo MH, Katona IM, Miller FW, Lachenbruch PA. Development of validated disease activity and damage indices for the juvenile idiopathic inflammatory myopathies: I. Physician, parent, and patient global assessments. Juvenile Dermatomyositis Disease Activity Collaborative Study Group. Arthritis Rheum. 1997 Nov;40(11):1976–83.

39. Lee KA, Hicks G, Nino-Murcia G. Validity and reliability of a scale to assess fatigue. Psychiatry Res. 1991 Mar;36(3):291–8. 10.1016/0165-1781(91)90027-m. PMID: 2,062,970.

40: Smith BW, Dalen J, Wiggins K, Tooley E, Christopher P, Bernard J. The brief resilience scale: assessing the ability to bounce back. Int J Behav Med. 2008;15(3):194–200. 10.1080/10705500802222972. PMID: 18,696,313.

41: Wolfe GI, Herbelin L, Nations SP, Foster B, Bryan WW, Barohn RJ. Myasthenia gravis activities of daily living profile. Neurology. 1999 Apr 22;52(7):1487–9. 10.1212/wnl.52.7.1487. PMID: 10,227,640.

42. Couper M, Tourangeau R, Conrad F, et al.: Evaluating the effectiveness of visual analog scales: A web experiment. Soc Sci Comput Rev 2006;24:227–245.

## Supplementary Information

Below is the link to the electronic supplementary material.

Supplementary file1 (PDF 483 KB)

## Data Availability

The datasets generated and/or analyzed during the current study are not publicly available but are available from the corresponding author upon reasonable request.

## Declarations

### Disclaimer

No part of this manuscript has been copied or published elsewhere, either in whole or in part.
