# Supplementary material for: Collating the voice of people with autoimmune diseases: Methodology for the Third Phase of the COVAD Studies
Source: Rheumatol Int. 2024 Apr 12;44(7):1233–44. doi: 10.1007/s00296-024-05562-z (PMC11178609; doi:10.1007/s00296-024-05562-z)
Supplement: Supplementary file 1 — (DOCX 62 KB) [file 296_2024_5562_MOESM1_ESM.docx]

**Supplementary File 1. Collating the Voice of People with Autoimmune Diseases (COVAD-3) Study Group Author List and Affiliations**

1. **Algeria**
2. **Dr** Chafia **Dahou Makhloufi** - professor in rheumatology, faculty of medicine. Bab El Oued University Hospital, Algiers, Algeria. [makhloufi-dahou@hotmail.com](mailto:makhloufi-dahou@hotmail.com). ORCID: 0000-0002-6523-9851
3. **Argentina**
4. **Dr** Leandro Gabriel **Ferreyra Garrott** - Rheumatologist. Sección de Reumatologia, Servicio de Clínica Médica. Hospital Italiano de Buenos Aires. [leandro.ferreyra@hospitalitaliano.org.ar](mailto:leandro.ferreyra@hospitalitaliano.org.ar). ORCID: 0000-0003- 3326-7628
5. **Armenia**
6. Dr Valentina **Vardanyan:** Yerevan State Medical University, Mikayelyan University Hospital, Yerevan, Armenia. ORCID: 0000-0002-8582-7837. [valentina.vardanyan@gmail.com](mailto:valentina.vardanyan@gmail.com)
7. **Australia**
8. **Dr** Vidya Sadanand **Limaye**- 1. Rheumatology Department, Royal Adelaide Hospital, Port Rd, Adelaide SA 5000, Australia. 2. Discipline of Medicine, University of Adelaide, North Tce, Adelaide SA 5000, Australia. ORCID: 0000-0002-8142-9532. [vidya.limaye@sa.gov.au](mailto:vidya.limaye@sa.gov.au)
9. **Dr** Nilesh **Srivastav**- University of Melbourne, Australia. ORCID: 0000-0002-1015-9280. [nilesh.srivastav@unimelb.edu.au](mailto:nilesh.srivastav@unimelb.edu.au).
10. **Bangladesh**
11. **Dr** A.T.M. Tanveer **Hasan**- Associate Professor of Rheumatology, Enam Medical College & Hospital, Bangladesh. [rousseau150.dmc@gmail.com](mailto:rousseau150.dmc@gmail.com) ORCID: 0000-0001-9809-839X
12. **Belarus**
13. Dr Marharyta **Volkava**: Belarusian Medical Academy of Post-Graduate Education, Minsk, Republic of Belarus. ORCID: 0000-0001-8572-9252. [margovolkova@gmail.com](mailto:margovolkova@gmail.com)
14. **Bhutan**
15. **Dr** Sonam **Yangchen** - Jigme Dorji Wangchuk National Referral Hospital. [sonamqos@gmail.com](mailto:sonamqos@gmail.com).
16. **Brazil**
17. **Dr** Dimitri **Luz Felipe da Silva -** University Santo Amaro; Hospital Israelita Albert Einstein. [dimitriluzfs@gmail.com](mailto:dimitriluzfs@gmail.com). ORCID: 0000-0003-0869-9330
18. **Dr** Odirlei Andre **Monticielo -** Division of Rheumatology, Department of Internal Medicine, Hospital de Clínicas de Porto Alegre, Universidade Federal Do Rio Grande Do Sul, Porto Alegre, Brazil. [omonticielo@gmail.com](mailto:omonticielo@gmail.com). ORCID: 0000-0003-0720-2097
19. **Bulgaria**
20. **Dr** Russka **Shumnalieva**: Department of Rheumatology, Clinic of Rheumatology, Medical University-Sofia, Bulgaria. [rshumnalieva@yahoo.com](mailto:rshumnalieva@yahoo.com) . ORCID: 0000-0003-2321-6536
21. **Dr** Rositsa Svetoslavova **Dacheva** - Diagnostic and Consultation Center UMHAT "St.Ivan Rilski", Sofia, Bulgaria. [rdacheva@gmail.com](mailto:rdacheva@gmail.com). ORCID: 0000-0002-5474-4131
22. **Cambodia**
    - - 1. **Dr Koy Rattanak:** Rheumatologist, Sonlak joint experts, Phnom Penh, Cambodia.

[Koyrattanak8787@gmail.com](mailto:Koyrattanak8787@gmail.com)

1. **Cameroon**
2. **Dr** Fernando **Kemta Lekpa** - 1 Department of Internal Medicine and Specialties, Faculty of Medicine and Pharmaceutical Sciences, University of Dschang, Dschang, Cameroon 2 Department of Internal Medicine, Douala General Hospital, Douala, Cameroon. [fklekpa@yahoo.fr](mailto:fklekpa@yahoo.fr). ORCID: 0000-0001-7592-5049
3. **Canada**
4. **Dr** Océane **Landon-Cardinal-** Division of Rheumatology, Centre hospitalier de l'Université de Montréal (CHUM); CHUM Research Center; Department of Medicine, Université de Montréal, Montréal, Québec, Canada. o.landoncardinal@gmail.com. [oceane.landon-cardinal@umontreal.ca](mailto:oceane.landon-cardinal@umontreal.ca). ORCID: 0000-0002-3361-6165
5. **Dr** Vanessa **Ocampo** - Scarborough health network. Kensington eye Institute. University of Toronto. [Vanessa.ocampo@medportal.ca](mailto:Vanessa.ocampo@medportal.ca). ORCID: 0000-0002-5705-1111
6. **Chile**
7. **Dr María Francisca Bozán Pérez** - Universidad de Chile/Hospital Clínico Universidad de Chile. [franbozan@yahoo.com](mailto:franbozan@yahoo.com). ORCID: [0000-0002-6109-1773](https://orcid.org/0000-0002-6109-1773)
8. **Dr Antonia Valenzuela**: Depto. de Reumatología e Inmunología Clínica, División de Medicina Interna, Pontificia Universidad Católica de Chile. [antonia.valenzuela@uc.cl](mailto:antonia.valenzuela@uc.cl) ORCID: [0000-0003-3357-9402](https://orcid.org/0000-0003-3357-9402)

**15) Hong Kong**

**1. Dr** Ho **So** - The Chinese University of Hong Kong. [hoso@cuhk.edu.hk](mailto:hoso@cuhk.edu.hk). ORCID: 0000-0001-7113-9390

**2. Dr Iris Tang:** Clinical Assistant Professor from the Division of Rheumatology and Clinical Immunology at the Department of Medicine at the University of Hong Kong, Special administrative regions of China. [tykiris@hku.hk](mailto:tykiris@hku.hk) ORCID: 0000-0002-1636-5384.

**3. Dr Edmund KM Wong:** Department of Medicine and Therapeutics, Prince of Wales Hospital, Hong Kong, Special administrative regions of China. [edmundkmwong@gmail.com](mailto:edmundkmwong@gmail.com) ORCID: 0009-0008-5628-4184.

**16) Colombia**

- - - 1. **Dr** Sebastian **Herrera Uribe** - Clínica Las Américas Auna – ARTMÉDICA. [sebastianherrerauribe@gmail.com](mailto:sebastianherrerauribe@gmail.com). ORCID: 0000-0001-7247-1846

**17) Croatia**

1. **Dr Ivan Padjen** - Division of Clinical Immunology and Rheumatology, Department of Internal Medicine, University Hospital Centre Zagreb, University of Zagreb School of Medicine. [ivan_padjen@yahoo.ca](mailto:ivan_padjen@yahoo.ca). ORCID: 0000-0002-9249-9325
2. **Dr Zeljka Kardum** - Department of Rheumatology, Clinical Immunology and Allergology, University Hospital Centre Osijek, J. Huttlera 4, 31000, Osijek, Croatia. School of Medicine, University J. J. Strossmayer Osijek, Huttlera 4, 31000, Osijek, Croatia. [zeljkakardum@gmail.com](mailto:zeljkakardum@gmail.com). ORCID: 0000-0002-6220-3685

**18) Cyprus**

1. **Dr Konstantinos Parperis** - University of Cyprus Medical School. [kparpe02@ucy.ac.cy](mailto:kparpe02@ucy.ac.cy). ORCID: 0000-0001-6009-0130

**19) Denmark**

1. **Dr Karen Schreiber** - 1Danish Hospital for Rheumatic Diseases, University of Southern Denmark, Sønderborg, Denmark. 2Institute of Regional Health Research (IRS), University of Southern Denmark, Odense, Denmark. 3. Thrombosis and Haemophilia, Kings Healthcare Partners, Guy's and St Thomas' NHS Foundation Trust, London, UK. [kschreiber@danskgigthospital.dk](mailto:kschreiber@danskgigthospital.dk). ORCID: 0000-0003-0660-7690

**20) Dominican Republic**

1. **Dr.** Jossiell **Then Báez**: MD, Hospital Metropolitano de Santiago (HOMS), Santiago, Dominican Republic. [Jossiellthen@gmail.com](mailto:Jossiellthen@gmail.com). ORCID: 0000-0002-8727-2855

**21) Egypt**

1. **Dr** Reem Hamdy Abdellatif **Mohammed**: Department of Rheumatology and Clinical Immunology and Rehabilitation School of Medicine Cairo University. [rmhamdy@yahoo.com](mailto:rmhamdy@yahoo.com). ORCID: 0000-0003-4994-7687
2. **Dr** Hala Mohamed **Lotfy** - Department of Pediatrics and pediatric rheumatology, Cairo University. [drhlotfy28@gmail.com](mailto:drhlotfy28@gmail.com) or [dr_hlotfy@yahoo.com](mailto:dr_hlotfy@yahoo.com). ORCID: 0000-0002-2090-925X
3. **Dr** Walaa **Abdel Rahman Saleh** - Rheumatology Department, Faculty of Medicine, Cairo University, Egypt. [walaaabdelrahman@kasralainy.edu.eg](mailto:walaaabdelrahman@kasralainy.edu.eg). ORCID: 0000-0002-6531-2692
4. **Dr** Mervat **Eissa** - Rheumatology Department, Kasr Alainy Medical School, Cairo University. [mervateissa@kasralainy.edu.eg](mailto:mervateissa@kasralainy.edu.eg). ORCID: 0000-0001-7999-3401
5. **Dr** Tamer **A. Gheita** - Rheumatology Department, Faculty of Medicine, Cairo University, Egypt. [gheitamer@hotmail.com](mailto:gheitamer@hotmail.com). ORCID: 0000-0002-1155-9729

**22) Equador**

**Dr Carlos Rios** - Ecuadorian Society of Rheumatology, Guayaquil, Ecuador. [criosacosta@gmail.com](mailto:criosacosta@gmail.com). ORCID: 0000-0003-0221-8276

**23) Estonia**

1. Dr Sandra **Meisalu**: East-Tallinn Central Hospital, Tallinn, Estonia. ORCID: [0000-0001-9307-089X](https://orcid.org/0000-0001-9307-089X). [sandra.meisalu@gmail.com](mailto:sandra.meisalu@gmail.com%20)

**24) Ethiopia**

1. **Dr** Becky Abdissa **Adugna:** Rheumatology Unit, Internal Medicine Department, Addis Ababa University, Addis Ababa, Ethiopia. [becky.abdissa_a@aau.edu.et](mailto:becky.abdissa_a@aau.edu.et). ORCID: 0000-0002-9740-4516

**25) France**

1. **Dr.** Margherita **Giannini**, Service de Physiologie et Explorations Fonctionnelles Musculaires, University Hospital of Strasbourg; Referral Centre for Rare Autoimmune Diseases, Strasbourg; UR3072, University of Strasbourg, France. [margherita.giannini85@gmail.com](mailto:margherita.giannini85@gmail.com). ORCID: 0000-0002-4834-5804
2. **Dr** Julien **Campagne-** Internal Medicine Department, University of Lorraine, 34, Cours Leopold, CS 25233, CEDEX, 54052 Nancy, France. ^2^Internal Medicine Department, University Hospital of Nancy, Rue du Morvan, CEDEX, 54511 Vandœuvre-Lès-Nancy, France. [julien.campagne@uneos.fr](mailto:julien.campagne@uneos.fr). ORCID: 0000-0002-6186-4334.
3. **Dr Alain Meyer-** Explorations fonctionnelles musculaires, Centre de Référence des maladies autoimmunes rares, Hôpitaux Universitaires de Strasbourg; UR 3072 Centre de Recherche en biomedecine, Université de Strasbourg; European referrence network ReCONNET. [alain.meyer7@gmail.com](mailto:alain.meyer7@gmail.com). [alain.meyer1@chru-strasbourg.fr](mailto:alain.meyer1@chru-strasbourg.fr)

**26) Germany**

1. **Dr** Hannah **Labinsky** - Department of Internal Medicine II, Rheumatology/Clinical Immunology, University of Würzburg, Wuerzburg, Germany. [labinsky_h@ukw.de](mailto:labinsky_h@ukw.de). ORCID: 0000-0001-5762-9182

**28) Greece**:

- - - 1. **Dr Alexandros Panagiotopoulos** - Rheumatology Unit, First Department of Propaedeutic Internal Medicine, Joint Academic Rheumatology Program, Laiko Hospital, Medical School, National & Kapodistrian University of Athens, 11527 Athens, Greece. ORCID: 0000-0003-3612-9394. [alej.panagiotopoulos@gmail.com](mailto:alej.panagiotopoulos@gmail.com).

**29) Guatemala**

1. **Dr. Andrea Bran Ordóñez**: Hospital Universitario Esperanza, Hospital El Pilar, PANLAR, AMIG. [dra.abranordonez@gmail.com](mailto:dra.abranordonez@gmail.com). ORCID: 0000-0002-8758-7021

**30) Hungary**

1. **Dr Melinda Nagy-Vincze**- Division of Clinical Immunology, Faculty of Medicine, University of Debrecen, Móricz Zsigmond út 22, Debrecen, H-4032, Hungary. [melinda.nagyvincze@gmail.com](mailto:melinda.nagyvincze@gmail.com) ORCID: [0000-0003-0316-3828](https://orcid.org/0000-0003-0316-3828)

**31) India**

1. **Dr Bhupen Barman** – Additional Professor and Head, Department of Medicine, All India Institute of Medical Sciences (AIIMS), Guwahati. [drbhupenb@gmail.com](mailto:drbhupenb@gmail.com). ORCID: 0000-0002-5433-1310
2. **Dr Yogesh Preet Singh**- Assistant Professor, Division of Rheumatology and Clinical Immunology, Department of General Medicine, Himalayan Institute of Medical sciences, Swami Rama University, Jolly Grant, Dehradun, Uttarakhand, India. [yogeshmann@gmail.com](mailto:yogeshmann@gmail.com). ORCID: 0000-0003-1258-0041
3. **Dr Arunkumar R Pande-** Consultant Endocrinologist, Lucknow Endocrine Diabetes and thyroid clinic, India. [drarunendocrine@gmail.com](mailto:drarunendocrine@gmail.com). ORCID: 0000-0002-9570-1509
4. **Praggya Yaadav:** Maharashtra Institute of Medical Sciences and Research, Latur, Maharashtra, India. ORCID: 0000-0003-4372-2540. [praggya1726@gmail.com](mailto:praggya1726@gmail.com)
5. **Dr Jasmine Parihar:** Assistant Professor of Neurology, All India Institute of Medical Science, New Delhi, India. ORCID: 0000-0002-5545-4304. [jasparihar@gmail.com](mailto:jasparihar@gmail.com)
6. **Dr Lakshmi M.R.**: Sanjay Gandhi Postgraduate Institute of Medical Sciences, Lucknow, India. [lakshmi5sncs@gmail.com](mailto:lakshmi5sncs@gmail.com)
7. **Manali Sarkar:** Mahatma Gandhi Mission Medical College, Navi Mumbai, Maharashtra, India. ORCID: 0000-0002-6732-2907. [manalisarkar12@gmail.com](mailto:manalisarkar12@gmail.com)

**32) Indonesia**

1. **Dr Suryo Anggoro Kusumo Wibowo**, Division of Rheumatology, Department of Internal Medicine, Cipto Mangunkusumo Hospital/Faculty of Medicine, Universitas Indonesia. ORCID: [0000-0002-0089-0504](about:blank). [dr.suryoipd@gmail.com](mailto:dr.suryoipd@gmail.com).

**33) Iraq**

1. **Dr Maryam Masoumi** - Clinical research and development center. [m.masoumiy@gmail.com](mailto:m.masoumiy@gmail.com). ORCID: 0000-0003-2635-2656.
2. **Dr Avin Maroof** - University of Kurdistan Hawlêr. [avinmaroof@gmail.com](mailto:avinmaroof@gmail.com). ORCID: 0000-0002-6554-5314.
3. **Dr Asal Adnan Redha** - MD, Rheumatology Unit, Department of Medicine, Baghdad Teaching Hospital, Medical City, Baghdad, Iraq. [asaladnan1987@gmail.com](mailto:asaladnan1987@gmail.com). ORCID: 0000-0001-9140-5860.

**34) Israel**

1. **Mr Or Aharonov**, Department of Gerontology, Faculty of Social Welfare and Health Science, University of Haifa, Haifa, Israel. ORCID: 0000-0003-4107-9045. [or1020@gmail.com](mailto:or1020@gmail.com)

**35) Italy**

1. Dr Marta **Mosca**: Rheumatology Unit, University of Pisa, Pisa . ORCID: 0000-0001-5937-4574. [marta.mosca@med.unipi.it](mailto:marta.mosca@med.unipi.it)
2. **Dr. Nicoletta Del Papa**, Unità operativa complessa (UOC) Reumatologia Clinica, ASST G. Pini-CTO, Milano, Italy. [nicoletta.delpapa@asst-pini-cto.it](mailto:nicoletta.delpapa@asst-pini-cto.it). ORCID: 0000-0001-8130-4150
3. **Dr. Atzeni Fabiola**, Rheumatology Unit, University of Messina, Messina, Italy. [atzenifabiola@hotmail.com](mailto:atzenifabiola@hotmail.com). ORCID: 0000-0002-5574-2939
4. **Dr Marco Sebastiani**, Rheumatology Unit, University of Modena and Reggio Emilia, Azienda Ospedaliero-Universitaria Policlinico di Modena, Via del Pozzo, 41125, Modena, Italy. [marco.sebastiani@unimore.it](mailto:marco.sebastiani@unimore.it). ORCID: 0000-0002-1294-6421
5. **Dr Franco Franceschini**, Department of Clinical and experimental sciences - University of Brescia; Rheumatology and Clinical Immunology Unit - ASST Spedali Civili of Brescia, Italy. [franco.franceschini@unibs.it](mailto:franco.franceschini@unibs.it). ORCID: 0000-0003-3678-6124
6. **Dr Pier Paolo Sainaghi**, Department of Translational Medicine, Università del Piemonte Orientale UPO, Novara, Italy; Division of Internal Medicine, Rheumatology Unit, CAAD (Center for Translational Research on Autoimmune and Allergic Disease) Maggiore della Carità, Italy. [pierpaolo.sainaghi@med.uniupo.it](mailto:pierpaolo.sainaghi@med.uniupo.it). ORCID: 0000-0001-8322-9158
7. **Dr Maria Giovanna Danielli**, Clinica Medica, Dipartimento di Scienze Cliniche e Molecolari, Università Politecnica delle Marche, via Tronto 10A, 60126 Ancona, Italy. [m.g.danieli@univpm.it](mailto:m.g.danieli@univpm.it). ORCID: 0000-0002-9608-2252
8. **Dr Vincenzo Venerito**- University of Bari, Department of Precision and Regenerative Medicine and Ionian Area, Rheumatology Unit, Bari, Italy. [vincenzo.venerito@gmail.com](mailto:vincenzo.venerito@gmail.com). ORCID: 0000-0002-9651-7295
9. **Dr. Alessandro Giollo:** Rheumatology Unit, University of Padua, Italy. [alessandro.giollo@unipd.it](mailto:alessandro.giollo@unipd.it) ORCID: 0000-0001-9355-7673
10. **Dr. Alessia Alluno:** University of L’Aquila – Department of Life, Health & Environmental Sciences; Internal Medicine and Nephrology Division, ASL 1 Avezzano-Sulmona-L'Aquila, San Salvatore Hospital, L'Aquila, Italy. [alessia.alunno82@gmail.com](mailto:alessia.alunno82@gmail.com). ORCID: 0000-0003-1105-5640
11. **Dr Devis Benfaremo** - Department of Clinical and Molecular Sciences, Marche Polytechnic University, Ancona, Italy. [d.benfaremo@staff.univpm.it](mailto:d.benfaremo@staff.univpm.it). ORCID: 0000-0002-9867-2360
12. **Dr Valentino Paci** - Medical Clinic and Internal Medicine Residency Programme, Department of Clinical and Molecular Sciences, Marche Polytechnic University & Department of Internal Medicine, Azienda Ospedaliero-Universitaria delle Marche, Italy. [valentinopaci@gmail.com](mailto:valentinopaci@gmail.com). ORCID: [0000-0002-9466-8460](https://orcid.org/0000-0002-9466-8460)
13. **Dr Giovanni Damiani** - 1 Department of Biomedical, Surgical and Dental Sciences, University of Milan, Milan, Italy. 2Italian Center of Precisione Medicine and Chronic Inflammation, University of Milan, Milan, Italy. [dr.giovanni.damiani@gmail.com](mailto:dr.giovanni.damiani@gmail.com). ORCID: 0000-0002-2390-6505
14. **Dr Elvis Hysa** - 1 Laboratory of Experimental Rheumatology and Academic Division of Clinical Rheumatology, Department of Internal Medicine, University of Genoa, Italy - IRCCS San Martino Polyclinic, Genoa, Italy. [elvis.hysa@gmail.com](mailto:elvis.hysa@gmail.com). ORCID: [0000-0002-6970-0983](https://orcid.org/0000-0002-6970-0983)
15. **Dr Matteo Lucchini** - Fondazione Policlinico Universitario Agostino Gemelli IRCCS, Rome Italy; Università Cattolica del Sacro Cuore, Rome, Italy. [matteo.lucchini@unicatt.it](mailto:matteo.lucchini@unicatt.it).
16. **Dr Alberto Lo Gullo** - UNIT OF RHEUMATOLOGY, ARNAS GARIBALDI CATANIA. ORCID: 0000-0003-4383-0314. [albertologullo@virgilio.it](mailto:albertologullo@virgilio.it).
17. **Dr Marco Fornaro**- University of Bari, Department of Precision and Regenerative Medicine and Ionian Area, Rheumatology Unit, Bari, Italy. [marco.fornaro@uniba.it](mailto:marco.fornaro@uniba.it) ORCID: [0000-0003-1716-7432](https://orcid.org/0000-0003-1716-7432)

**36) Japan**

1. **Dr Akira Yoshida** - Department of Allergy and Rheumatology, Nippon Medical School Graduate School of Medicine. [a-yoshida@nms.ac.jp](mailto:a-yoshida@nms.ac.jp). ORCID: 0000-0003-3590-1637
2. **Dr Saori Abe** - University of Tsukuba. [ori86114@gmail.com](mailto:ori86114@gmail.com). ORCID: 0000-0003-2793-5589
3. **Dr Ran Nakashima (Sasai)**- Department of Rheumatology and Clinical Immunology, Graduate School of Medicine, Kyoto University, 54 Shogoin-Kawahara-cho, Sakyo-ku, Kyoto 606-8507, Japan [ranran@kuhp.kyoto-u.ac.jp](mailto:ranran@kuhp.kyoto-u.ac.jp)

**37) Jordan**

1. **Dr Fatima Alnaimat** - University of Jordan. [f.naimat@ju.edu.jo](mailto:f.naimat@ju.edu.jo) AND [falnaimat@yahoo.com](mailto:falnaimat@yahoo.com). ORCID: 0000-0002-5574-2939

**38) Kazakhstan**

1. **Dr Gulzhan Trimova** - MD, PhD, Department of Clinical Subjects, al-Farabi Kazakh National Medical University, Almaty, Kazakhstan. City Rheumatology Center, Almaty, Kazakhstan. [trimova@gmail.com](mailto:trimova@gmail.com). ORCID: 0000-0001-8130-4150
2. **Dr Dana Bekaryssova** - South Kazakhstan Medical Academy: Shymkent, KZ. [bekaryssova.da@gmail.com](mailto:bekaryssova.da@gmail.com). ORCID: 0000-0002-9651-7295

**39) Kenya**

1. **Dr Eugene kalman Genga** - Lecturer at University of Nairobi. [eugenekalman@gmail.com](mailto:eugenekalman@gmail.com). ORCID: 0000-0001-5866-7105

**41) Libya**

1. **Dr Soad Salem Hashad** - Tripoli children hospital /university of Tripoli. [soadhashad@hotmail.com](mailto:soadhashad@hotmail.com). ORCID: 0000-0003-0500-7458
2. Dr Laila **Ayoub**: ^1^Faculty of Medicine, University of Tripoli, Rheumatology, Tripoli, Libya; ^2.^ Tripoli Central Hospital, Internal medicine, Tripoli, Libya. ORCID: [0000-0003-0381-2197](about:blank). [laila_ayoub21@yahoo.ca](mailto:laila_ayoub21@yahoo.ca)

**42) Malaysia**:

1. **Dr Syahrul Sazliyana Shaharir** - Department of Medicine, Faculty of Medicine, Universiti Kebangsaan Malaysia. [sazliyana@ukm.edu.my](mailto:sazliyana@ukm.edu.my). ORCID: 0000-0002-9068-8114
2. **Dr Tan Chou Luan** - 1. Rheumatology Unit, Department of Medicine, Hospital Sultanah Bahiyah, Malaysia 2. Rheumatology Unit, Department of Medicine, Hospital Putrajaya, Malaysia. [chouluan@gmail.com](mailto:chouluan@gmail.com). ORCID: 0000-0002-9859-5385

**43) Malta**

1. **Dr Rosalie** **Magro**: ^1^Rheumatology Department, Mater Dei Hospital, Tal-Qroqq, Msida, MSD, 2090, Malta; ^2^Faculty of Medicine and Surgery, University of Malta, Msida, Malta. ORCID: 0000-0001-8486-8410. [rosaliemagro@gmail.com](mailto:rosaliemagro@gmail.com)

**44) Mexico**

1. **Dr Erick Adrian Zamora Tehozol:** Centro Médico Pensiones, Mexico . [erick.zamorat@outlook.com](mailto:erick.zamorat@outlook.com). ORCID: [0000-0002-7888-3961](https://orcid.org/0000-0002-7888-3961)
2. **Dr Ignacio García-De La Torre-** Universidad de Guadalajara. Department of Immunology and Rheumatology, Hospital General de Occidente. Guadalajara, Jal. México. [igdlt@aol.com](mailto:igdlt@aol.com). ORCID: 0000-0002-9261-678X
3. **Dr. Iris J. Colunga‑Pedraza**: Hospital Universitario UANL monterrey Mexico, Mexico. [iriscolunga@hotmail.com](mailto:iriscolunga@hotmail.com). ORCID: 0000-0002-2786-5843
4. **Dr.** **Javier Merayo-Chalico**: Department of Immunology and Rheumatology, Instituto Nacional de Ciencias Médicas y Nutrición “Salvador Zubirán”, Mexico City, Mexico. [oyarem@hotmail.com](mailto:oyarem@hotmail.com). ORCID: 0000-0002-5870-0523
5. **Dr Deshire Alpizar-Rodriguez** - Research Unit, Mexican College of Rheumatology. [deshire_alpizar@hotmail.com](mailto:deshire_alpizar@hotmail.com). ORCID: 0000-0002-6930-0517
6. **Dr Miguel Angel Saavedra Salinas** - Research Division, Hospital de Especialidades Dr. Antonio Fraga Mouret, CMN La Raza, IMSS, Mexico City. [miansaavsa@gmail.com](mailto:miansaavsa@gmail.com). ORCID: 0000-0003-0687-9944

**45) Moldova**

1. **Dr Victoria Sadovici-Bobeica** - State University of Medicine and Pharmacy “Nicolae Testemitanu”. [Victoria.sadovici-bobeica@usmf.md](mailto:Victoria.sadovici-bobeica@usmf.md). ORCID: 0000-0003-1803-6960.

**46) Mongolia**

1. **Dr Zulgerel Dandii** - Mongolian national University of Medical sciences. [zulgerel@mnums.edu.mn](mailto:zulgerel@mnums.edu.mn).

**47) Morocco**

1. **Dr Ihsane Hmamouchi-** 1. Health Sciences Research Centre (CReSS), Morocco. 2.Faculty of Medicine, International University of Rabat (UIR), Morocco. [ihsane.hmamouchi@gmail.com](mailto:ihsane.hmamouchi@gmail.com). ORCID: 0000-0003-4402-5034.

**48) Nepal**

1. **Dr Binit Vaidya**- Department of Rheumatology, National Centre for Rheumatic Diseases, Ratopul, Kathmandu, Nepal. [drbinitvaidya@gmail.com](mailto:drbinitvaidya@gmail.com) ORCID: [0000-0002-4840-8924](https://orcid.org/0000-0002-4840-8924)

**49) Netherlands**

- - - 1. **Dr Luis Fernando Perez Garcia:** Department of Rheumatology, Erasmus Medical Center, Rotterdam, Netherlands. ORCID: 0000-0002-8958-9493 [l.perez@erasmusmc.nl](mailto:l.perez@erasmusmc.nl)

**51) Nicaragua**

1. **Dr** **Idania Calixta Escalante Mendoza**: Oscar Danilo Rosales Arguello School Hospital. [idaniaescalante@ufm.edu](mailto:idaniaescalante@ufm.edu). ORCID: 0000-0003-0156-8098
2. **Dr Christian Mauriel Uriarte Hernandez** - Hospital Vivian Pellas. Clínica de Reumatología. [drchristianuriarte@hotmail.com](mailto:drchristianuriarte@hotmail.com). ORCID: 0009-0009-3424-7961

**52) Niger**

- - - 1. **Dr** Moussa Elh Ibrahim **Fanata**: Head of the Rheumatology Department, Niamey National Hospital, Niger. [fanatamoussa@yahoo.fr](mailto:fanatamoussa@yahoo.fr)

**53) Nigeria**

1. **Dr** Uyiekpen **Ima-Edomwonyi-** Consultant, Department of Internal Medicine, Lagos University Teaching Hospital, Lagos, Nigeria. [get2uyi@gmail.com](mailto:get2uyi@gmail.com) ORCID: 0000-0001-7651-5025
2. **Dr** Ibukunoluwa **Dedeke**- Consultant Rheumatologist and Physician, University College Hospital Ibadan, Oyo state Nigeria; Associate lecturer, College of Medicine, University of Ibadan. [dr.iadedeke@gmail.com](mailto:dr.iadedeke@gmail.com). ORCID: [0000-0002-6494-0654](https://orcid.org/0000-0002-6494-0654)
3. **Dr** Airenakho **Emorinken** - Rheumatology Division, Department of Internal Medicine, Irrua specialist Teaching Hospital, Irrua, Edo State. [emosairen@gmail.com](mailto:emosairen@gmail.com) ORCID: [0000-0002-3306-572X](https://orcid.org/0000-0002-3306-572X)
4. Dr Henry Madu **Nwankwo** - Department of internal medicine, Faculty of Medicine, Nnamdi Azikiwe University, Awka, Nnewi Campus Anambra State Nigeria. [hm.nwankwo@unizik.edu.ng](mailto:hm.nwankwo@unizik.edu.ng). ORCID: [0000-0002-5679-7093](https://orcid.org/0000-0002-5679-7093)
5. **Dr** Hakeem Babatunde **Olaosebikan** - Consultant Rheumatologist, Lagos State University College of Medicine/Lagos State University Teaching Hospital, Ikeja, Lagos, Nigeria. +2348035751154. [hakeemolaosebikan37@gmail.com](mailto:hakeemolaosebikan37@gmail.com). ORCID: 0000-0003-4004-0093
6. **Dr** Celestine **Okwara**: Rheumatology, University of Nigeria Teaching Hospital, Enugu, Nigeria. [celestine.okwara@unn.edu.ng](mailto:celestine.okwara@unn.edu.ng). ORCID: 0000-0002-7727-5657
7. **Dr Tralagba Uchechukwu** - University of Port Harcourt Teaching Hospital, Port Harcourt, Rivers State, Nigeria. [okwaracc@yahoo.com](mailto:okwaracc@yahoo.com).

**54) Oman**

1. **Dr** Batool **Hassan** - Dr Kowther Hassan Dr Talal Al Lawati. [bhassan@squ.edu.om](mailto:bhassan@squ.edu.om). ORCID: 0000-0002-5954-0189

**55) Pakistan**

1. Dr. Uzma **Rasheed** - Pakistan Institute of Medical Sciences Islamabad, Shaheed Zulfiqar Ali Bhutto Medical University. ORCID: 0009-0001-5122-0703. [uzma_sheikh11@yahoo.com](mailto:uzma_sheikh11@yahoo.com)
2. **Dr Babur Salim-** Rheumatology Department, Fauji Foundation Hospital, Rawalpindi, Pakistan. Orcid ID: 0000-0001-8430-9299. [babursalim@yahoo.com](mailto:babursalim@yahoo.com)

**56) Paraguay**

1. **Dr.** Nelly **Colman Mc Leod**: Universidad Nacional de Asunción, Hospital de Clínicas de la Facultad de Ciencias Médicas, Departamento de Reumatología. Asunción, Paraguay. [dra.nellycolman@gmail.com](mailto:dra.nellycolman@gmail.com). ORCID: 0000-0001-8709-6567

**57) Peru**

1. **Dr** Manuel Francisco **Ugarte-Gil**- Grupo Peruano de Estudio de Enfermedades Autoinmunes Sistémicas, Universidad Cientifica del Sur, Lima, Peru & Rheumatology Department, Hospital Nacional Guillermo Almenara Irigoyen, EsSalud, Lima, Peru. [manuel_ugarte@yahoo.com](mailto:manuel_ugarte@yahoo.com). ORCID: 0000-0003-1728-1999
2. Dr. Victor **Pimentel-Quiroz**: Hospital Nacional Guillermo Almenara Irigoyen/Universidad Científica del Sur. [victorpq4@gmail.com](mailto:victorpq4@gmail.com). ORCID: 0000-0002-3638-7054

**58) Philippines**

1. Dr Lisa S **Traboco** 1. St Luke's Medical Center-Global City, 2. University of the Philippines-Manila, MIU. [lisatraboco@gmail.com](mailto:lisatraboco@gmail.com). ORCID: 0000-0002-1952-7879

**59) Poland**

1. **Dr** Joanna **Makowska** - Department of Rheumatology, Medical University of Lodz. [joanna.makowska@umed.lodz.pl](mailto:joanna.makowska@umed.lodz.pl). ORCID: 0000-0003-2036-375X
2. **Dr** Aleksandra **Opinc-Rosiak** - Department of Rheumatology, Medical University of Lodz, Zeromskiego 113, 90-549 Lodz, Poland. [aleksandra.opinc@umed.lodz.pl](mailto:aleksandra.opinc@umed.lodz.pl). ORCID: 0000-0002-7881-5731
3. **Dr** Marcin **Milchert** - Department of Internal Medicine, Rheumatology, Diabethology, Geriatrics and Clinical Immunology of Pomeranian Medical University in Szczecin. [marcmilc@hotmail.com](mailto:marcmilc@hotmail.com). ORCID: 0000-0002-0943-8768

**60) Portugal**

- - - 1. **Dr Luís Sousa Inês** - (1) Rheumatology Department, Centro Hospitalar Universitário de Coimbra EPE, Coimbra, Portugal. (2) Faculty of Health Sciences, Universidade da Beira Interior. Covilhã, Portugal. [luisines@gmail.com](mailto:luisines@gmail.com). ORCID: 0000-0003-3172-3570

**61) Qatar**

**Dr Samar Al Emadi:** Medicine Depar tment, Rheumatology Section, Hamad Medical Corporation, Doha, Qatar. ORCID: 0000-0001-7942-4831. [salemadi@hamad.qa](mailto:salemadi@hamad.qa)

**62) Romania**

1. **Dr Cristina Alexandru** - Internal Medicine and Rheumatology Department, Dr. Ion Cantacuzino Clinical Hospital,011437 Bucharest, Romania. [crista.maria.alexandru@gmail.com](mailto:crista.maria.alexandru@gmail.com). ORCID: 0000-0001-7053-7530
2. **Dr** Anca **Bobircă** - Department of Internal Medicine and Rheumatology University of Medicine and Pharmacy Carol Davila Bucharest. Hospital Dr I Cantacuzino. [Anca.bobirca@umfcd.ro](mailto:Anca.bobirca@umfcd.ro). ORCID: 0000-0002-6662-7354
3. **Dr** Claudia **Cobilinschi** – 1. Department of Rheumatology and Internal Medicine, Sf Maria Clinical Hospital Bucharest, 011172, Bucharest, Romania; 2. Department of Rheumatology and Internal Medicine, Carol Davila University of Medicine and Pharmacy, 050474 Bucharest, Romania. [claudiadeaconu1@yahoo.com](mailto:claudiadeaconu1@yahoo.com) ORCID: 0000-0002-9117-2187

**63) Russian Federation**

1. **Dr** Margarita Aleksandrovna **Gromova**, Pirogov Russian National Research Medical University (RNRMU), Moscow, Russian Federation. [margarita-gromov@mail.ru](mailto:margarita-gromov@mail.ru) ORCID: 0000-0002-3757-058X

**64) Rwanda**

- - - 1. **Dr Mubirigi Alexandre:** Pediatrician, Kacyiru Police Hospital, Kigali, Rwanda. [mubiralex@gmail.com](mailto:mubiralex@gmail.com) ORCID: 0000-0003-3413-9855.

**65) Saudi Arabia**

1. **Dr Lina El Kibbe**- Division of Rheumatology, Department of Internal Medicine, Specialized Medical Center, Riyadh, Saudi Arabia. [linakibbe@gmail.com](mailto:linakibbe@gmail.com). ORCID: 0000-0003-1710-9996
2. **Dr Hussein Mohammed Halabi**: Department of medicine, section of rheumatology, King Faisal specialist hospital and research center, Jeddah, Saudi Arabia. [mfasel@gmail.com](mailto:mfasel@gmail.com). ORCID: 0000-0002-5174-8292

**66) Serbia**

1. **Dr Rada Miskovic** - Clinic of Allergy and Immunology, University Clinical Centre of Serbia; Faculty of Medicine, University of Belgrade. [rada_delic@hotmail.com](mailto:rada_delic@hotmail.com). ORCID: 0000-0003-0982-2572

**67) Singapore**

1. **Dr Anindita Santosa:** Division of Rheumatology, Changi General Hospital, Singapore. ORCID: 0000-0002-7320-4552. [anindita.santosa@singhealth.com.sg](mailto:anindita.santosa@singhealth.com.sg)
2. **Dr Desmond Chua**: Tan Tock Seng Hospital, Singapore. [desmondchuacg@gmail.com](mailto:desmondchuacg@gmail.com)

**68) Spain**

1. **Dr Cristiana Sieiro Santos**: Rheumatology Department, Complejo Asistencial Universitario de León, León, Spain. ORCID: 0000-0003-0889-9877. [cristysieirosantos@gmail.com](mailto:cristysieirosantos@gmail.com)
2. **Dr Jesús Loarce-Martos**: Ramón y Cajal University Hospital, Madrid, Spain. [jesus.loarce@gmail.com](mailto:jesus.loarce@gmail.com). ORCID: 0000-0003-1352-9539
3. **Dr Sergio Prieto-González**: Department of Internal Medicine and Autoimmune Diseases. Hospital Clínic of Barcelona. IDIBAPS. University of Barcelona, Spain. [sprieto@clinic.cat](mailto:sprieto@clinic.cat). ORCID: 0000-0002-4900-1675
4. **Dr Juan Molina-Collada** - Hospital General Universitario Gregorio Marañón, Madrid, Spain. [molinacolladajuan@gmail.com](mailto:molinacolladajuan@gmail.com). ORCID: 0000-0001-5191-7802

**69) Sri Lanka**

1. **Dr Chathurika Lakmini Dandeniya** - Senior Lecturer at the Department of Medicine, Faculty of Medicine, University of Peradeniya and Honorary Consultant Rheumatologist at the University Teaching Hospital, Peradeniya. [chathurika.dandeniya@yahoo.com](mailto:chathurika.dandeniya@yahoo.com). ORCID: 0000-0001-8857-1366

**70) Sudan**

1. **Dr Ahmed Seri Ibrahim Mohamed** - Clinical Immunology and Allergy center, Royal Care International Hospital. [dr.seri@outlook.com](mailto:dr.seri@outlook.com). ORCID: 0000-0002-6516-5450

**72) Switzerland**

1. **Dr Raphael Micheroli** - Department of Rheumatology, University Hospital Zurich, University of Zurich, Zurich, Switzerland. [raphael.micheroli@usz.ch](mailto:raphael.micheroli@usz.ch). ORCID: [0000-0002-8918-7304](https://orcid.org/0000-0002-8918-7304)
2. **Dr Lisa Christ** - Department of Rheumatology and Immunology, Inselspital, Bern University Hospital, University of Bern, Bern, Switzerland. [lisa.christ@insel.ch](mailto:lisa.christ@insel.ch). ORCID: 0000-0002-7358-2051
3. **Dr Oliver Distler** - Department of Rheumatology, University Hospital Zurich, University of Zurich, Switzerland. [oliver.distler@usz.ch](mailto:oliver.distler@usz.ch). ORCID: 0000-0002-0546-8310

**73) Syria**

1. **Dr Nada Alchama** - Consultant rheumatologist in Syria.IbN alnafis hospital. [Nada_alshamaa@yahoo.com](mailto:Nada_alshamaa@yahoo.com). ORCID: 0000-0001-6305-4581

**74) Taiwan**

1. **Dr Yi-Ming Chen**- Division of Allergy, Immunology, and Rheumatology, Department of Internal Medicine, Taichung Veterans General Hospital, Taichung, Taiwan; Department of Post-Baccalaureate Medicine, College of Medicine, National Chung Hsing University, Taichung, Taiwan. [ymchen1@vghtc.gov.tw](mailto:ymchen1@vghtc.gov.tw). ORCID: 0000-0001-7593-3065
2. **Dr Chih-Wei Tseng** - 1. Division of Allergy, Immunology and Rheumatology, Department of Internal Medicine, Taichung Veterans General Hospital, Taichung, Taiwan. 2. Department of Public Health, College of Medicine, National Cheng Kung University, Tainan, Taiwan. [deutschwewe@gmail.com](mailto:deutschwewe@gmail.com); [cwtseng@vghtc.gov.tw](mailto:cwtseng@vghtc.gov.tw). ORCID: 0000-0002-5948-7306

**75) Thailand**

1. **Dr Phonpen Akarawatcharangura**- Department of Medicine, Queen Savang Vadhana Memorial Hospital, Chonburi, Thailand. [phonpen@msn.com](mailto:phonpen@msn.com) ORCID: [0009-0009-7611-9992](https://orcid.org/0009-0009-7611-9992)
2. **Dr. Wanruchada Katchamart:** Division of Rheumatology, Department of Medicine, Faculty of Medicine Siriraj Hospital, Mahidol University, Bangkok, Thailand. [wanda.katchamart@gmail.com](mailto:wanda.katchamart@gmail.com) , [wanruchada.kat@mahidol.ac.th](mailto:wanruchada.kat@mahidol.ac.th). ORCID 0000-0002-8952-5967

**76) Tunisia**:

- - - 1. **Dr Wafa Hamdi** - Department of Rheumatology Kassab Ksar Said Institute Tunis Tunisia- Faculty of medecine of Tunis - Tunis El Manar University. ORCID: 0000-0003-3045-4831. [wafahamdi6@yahoo.fr](mailto:wafahamdi6@yahoo.fr) ; [wafa.hamdi@fmt.utm.tn](mailto:wafa.hamdi@fmt.utm.tn).

**77) Turkey**

1. **Dr Döndü Üsküdar Cansu**: Prof.Dr (Division of Rheumatology, Department of Internal Medicine,Eskişehir Osmangazi University School of Medicine: Eskişehir, TR). [ducansu@hotmail.com](mailto:ducansu@hotmail.com). ORCID: 0000-0001-6543-3905.
2. **Dr Reşit Yıldırım**- Osmangazi University, Division of Rheumatology. [celeng18@gmail.com](mailto:celeng18@gmail.com). ORCID: 0000-0003-4040-0212
3. **Dr Tugba Izci Duran** - Denizli State Hospital. [drtugbaizciduran@gmail.com](mailto:drtugbaizciduran@gmail.com). ORCID: 0000-0003-4428-9873
4. **Dr Neslihan Gokcen** - Kocaeli university faculty of medicine. [drngokcen@hotmail.com](mailto:drngokcen@hotmail.com). ORCID: 0000-0003-3022-493X

**78) Ukraine**

1. **Dr** **Myroslava Kulyk**: Bogomolets National Medical University, Internal Medicine Department No 2, Kyiv, Ukraine. ORCID: 0000-0002-7695-9977. [myroslavakulyk@gmail.com](mailto:myroslavakulyk@gmail.com)

**79) United Arab Emirates**

1. **Dr Nesreen Mohamed Ismail Moustafa** - Rheumatology Department- Suez Canal University, Ismailia, Egypt. [tasneemge@yahoo.com](mailto:tasneemge@yahoo.com). ORCID: 0000-0002-4900-1675

**80) United Kingdom**

1. **Dr John D Pauling**: 1. Royal National Hospital for Rheumatic Diseases (at Royal United Hospitals), Upper Borough Walls, Bath, BA1 1RL, UK. 2. Department of Pharmacy and Pharmacology, University of Bath, Bath, UK. [JohnPauling@nhs.net](mailto:JohnPauling@nhs.net) ORCID: [0000-0002-2793-2364](https://orcid.org/0000-0002-2793-2364)
2. **Dr Tania Gudu** - 1- Rheumatology Department, Ipswich Hospital, ESNEFT, Ipswich, UK 2- Psychology Department, EEPRU, University of Essex, Colchester, UK. [taniagudu@gmail.com](mailto:taniagudu@gmail.com). ORCID: 0000-0002-8973-323X
3. **Dr Valentina Pucino** - Institute of Inflammation and Ageing (Birmingham), Kennedy Institute of Rheumatology (Oxford). [valentina.pucino@gmail.com](mailto:valentina.pucino@gmail.com). ORCID: 0000-0002-7683-4568.
4. **Dr Melanie Sloan** - Department of public health, university of Cambridge. [mas229@medschl.cam.ac.uk](mailto:mas229@medschl.cam.ac.uk). ORCID: 0000-0001-8153-9064
5. **Dr Sarah Dyball** – 1. Centre for Epidemiology Versus Arthritis, Division of Musculoskeletal and Dermatological Sciences, Faculty of Biology, Medicine and Health, University of Manchester, Manchester Academic Health Science Centre, Manchester, UK. 2. Kellgren Centre for Rheumatology, Manchester University Hospitals NHS Foundation Trust, Manchester, UK. [sarah.dyball@manchester.ac.uk](mailto:sarah.dyball@manchester.ac.uk). ORCID: 0000-0001-6201-7391.
6. **Dr James Lilleker** - ^1.^ Centre for Musculoskeletal Research, Division of Musculoskeletal and Dermatological Sciences, School of Biological Sciences, Faculty of Biology, Medicine and Health, Manchester Academic Health Science Centre, The University of Manchester, Manchester, UK. ^2^ Neurology, Manchester Centre for Clinical Neurosciences, Northern Care Alliance NHS Foundation Trust, Salford, UK. [james.lilleker@manchester.ac.uk](mailto:james.lilleker@manchester.ac.uk). ORCID: 0000-0002-9230-4137
7. **Dr Aurelie Najm**: School of Infection and Immunity, College of Medical Veterinary and Life Sciences, University of Glasgow, Glasgow, UK. ORCID: 0000-0002-6008-503X. [*Aurelie*.*Najm*@glasgow.ac.uk](mailto:Aurelie.Najm@glasgow.ac.uk)
8. **Dr** **Arvind Nune**- Southport and Ormskirk Hospital NHS Trust, Southport, PR8 6PN, UK. [Arvind.Nune@merseywestlancs.nhs.uk](mailto:Arvind.Nune@merseywestlancs.nhs.uk)  ORCID: [0000-0002-3849-614X](https://orcid.org/0000-0002-3849-614X)

**81) United States of America**

1. **Dr Aarat Patel**: Bon Secours Rheumatology Center and Division of Pediatric Rheumatology, Department of Pediatrics, University of Virginia, Bon Secours Mercy Health, USA. [aarat_patel@bshsi.org](mailto:aarat_patel@bshsi.org). ORCID: 0000-0002-9756-2274
2. **Dr Shikha Singla** – Medical College of Wisconsin. [shikha.singla@gmail.com](mailto:shikha.singla@gmail.com). ORCID: 0000-0002-5540-8850
3. **Dr. Mazen Dimachkie:** Department of Neurology, SOM-Kansas City Associate Director, Associate Director of the Institute for Neurological Discoveries, SOM-Kansas City, Neurology Clinical Director, Director, Neuromuscular Division, SOM-Kansas City, Neurology. [mdimachkie@kumc.edu](mailto:mdimachkie@kumc.edu)

**82) Venezuela**

1. **Dr. Yurilís Fuentes-Silva**: Departamento de Medicina, Universidad de Oriente, Ciudad Bolívar, Venezuela. [yurilisfuentes@gmail.com](mailto:yurilisfuentes@gmail.com). ORCID: 0000-0002-5915-769X

**83) Vietnam**

- - - 1. **Dr Bich Ngoc Nguyen:** Bach Mai Hospital, Hanoi, Vietnam, Karolinska University Hospital. [nguyenngocbichnt36bm@gmail.com](mailto:nguyenngocbichnt36bm@gmail.com)

**84) Zambia**

**Dr Panganani** **Njobvu**: University of Lusaka, School of Medicine and Levy Mwanawasa Teaching hospital, Lusaka, Zambia. ORCID: 0000-0002-1432-0836. Email address: [pnjobvu@unilus.ac.zm](mailto:pnjobvu@unilus.ac.zm)

**85) Honduras**

1. **Dr. Hugo Alonzo**- Jefe del Departamento de Medicina Interna en Hospital de Especialidades del Seguro Social en Tegucigalpa, Honduras alonzohugo@yahoo.com
2. **Dr. Carlos Benito Santiago Pastelin**- Médico especialista, Instituto Hondureño del Seguro Social, Honduras ?

**86) Panama**

1. **Dr. Generoso Guerra Bautista**: Centro de Investigación Marbella, Paitilla Panamá, Panamá generosoguerra@icloud.com
2. **Dr. Enrique Julio Giraldo Ho**: Rheumatologist, Universidad de Panamá, República de Panamá

**Patient research partners**

**1. Paula Jordan**: Honorary general secretary, Myositis UK. [prjjordan@icloud.com](mailto:prjjordan@icloud.com)

**2. Kirtida Oza:** Co-founder Director, Sjogren’s India, Ahmedabad, India. [kirtidaoza@gmail.com](mailto:kirtidaoza@gmail.com)

**3. Dr. Ingrid De Groot:** Chair Dutch Myositis Working Group, Patient Association for Neuromuscular Diseases, Baarn, The Netherlands. E-mail: ingrid.de.groot@upcmail.nl
