# Supplementary material for: Correction to: Collating the voice of people with autoimmune diseases: Methodology for the third phase of the COVAD studies
Source: Rheumatol Int. 2024 Oct 14;44(12):3157–62. doi: 10.1007/s00296-024-05692-4 (PMC11618184; doi:10.1007/s00296-024-05692-4)
Supplement: Supplementary file 1 — Supplementary file1 (PDF 483 KB) [file 296_2024_5692_MOESM1_ESM.pdf]

Collating the Voice of people living with Autoimmune Diseases (CoVAD 3) Surv

The CoVAD team comprises a group of physician-scientists undertaking a project to determine patients' subjective well-being. By assessing these, we aim to understand your life holistically including factors influencing life satisfaction. These answers will help physicians understand the healthcare interventions needed to improve the lives of people living with chronic diseases.

If you are over 18 years of age, we would be grateful for fifteen minutes of your precious time to respond to this survey. You may fill out the survey at once or exit and re-enter the survey to complete it in parts at your convenience.
Patients with Myasthenia gravis, autoimmune or rheumatic disease and healthy individuals can take this survey. If you are a patient with an autoimmune or rheumatic disease, we encourage you to ask the healthy members of your family and friends to take this survey as well.
The data collected from this survey will be used for publication in peer-reviewed journals.
This survey was approved by the Institutional Review Board (IRB) or ethics committee (SGPGIMS, Lucknow) which governs the appropriateness of the conduct of this survey.
Your responses will be kept confidential and anonymous and will be stored securely with the Chief Investigator of the study. Any responses you provide will be anonymized, so that neither the research team nor additional respondents will know which is yours.
If you have any concerns or questions, please write to covadstudy@gmail.com or Dr. V Agarwal MD, DM at vikasagr@yahoo.com.

By participating in this study, you are agreeing to provide the most honest answers you can.
By clicking "Next" you are consenting to the conditions described above.

Disease Information

* 1. Has a doctor ever diagnosed you with any of the following rheumatic conditions? (please check the box for all that apply)

- [ ] I do not have any rheumatic disease
- [ ] I am not sure if I have a rheumatic disease yet, but I have symptoms under investigation
- [ ] Ankylosing spondylitis or Axial spondyloarthropathy
- [ ] Anti-Phospholipid syndrome (APS)
- [ ] Anti-Synthetase Syndrome (ASyS)
- [ ] Behcet’s disease
- [ ] Discoid lupus
- [ ] Familial Mediterranean fever
- [ ] Giant cell arteritis
- [ ] Gout
- [ ] IBD-associated arthritis (Arthritis related to Inflammatory Bowel Diseases such as Crohn’s disease or Ulcerative colitis): Sometimes known as Enteropathic arthritis
- [ ] IgG4 disease
- [ ] Juvenile idiopathic arthritis
- [ ] Mixed connective tissue disease (MCTD)
- [ ] Myositis
- [ ] Overlap myositis with lupus or Sjögren’s or systemic sclerosis or rheumatoid arthritis (syndrome comprising of a combination of myositis and one or more of the other mentioned diseases)
- [ ] Osteoarthritis
- [ ] Polymyalgia rheumatica (PMR)
- [ ] Psoriatic arthritis
- [ ] Pseudogout/CPPD (Calcium pyrophosphate deposition disease)
- [ ] Reactive arthritis
- [ ] Rheumatoid arthritis
- [ ] Sarcoidosis
- [ ] Sjögren’s syndrome
- [ ] Systemic lupus erythematosus (SLE)
- [ ] Systemic sclerosis (Scleroderma)
- [ ] Takayasu’s arteritis
- [ ] Undifferentiated connective tissue disease (UCTD)
- [ ] Vasculitis
- [ ] Another rheumatic disease (please specify) -

____

* 2. Has a doctor ever diagnosed you with any of the following autoimmune conditions? (please check the box for all that apply)

- ☐ I do not have any other autoimmune disease (besides the diseases listed in the previous question)
- ☐ I am not sure if I have any autoimmune disease yet, but I have symptoms under investigation.
- ☐ Alopecia areata
- ☐ Atopic dermatitis
- ☐ Celiac sprue
- ☐ Chronic inflammatory demyelinating polyradiculoneuropathy (CIDP)
- ☐ Crohn’s disease
- ☐ Grave’s disease (hyperthyroidism)- Reverse Hyperthyroid/Overactive Thyroid
- ☐ Hashimoto’s disease (hypothyroidism)- Reverse Hypothyroid/Underactive Thyroid
- ☐ Hemolytic anemia
- ☐ Idiopathic thrombocytopenic purpura (ITP)
- ☐ Morphea
- ☐ Multiple sclerosis
- ☐ Myasthenia gravis
- ☐ Pernicious anemia
- ☐ Psoriasis
- ☐ Type 1 diabetes mellitus
- ☐ Ulcerative colitis
- ☐ Autoimmune Hepatitis
- ☐ Uveitis
- ☐ Vitiligo
- ☐ Another autoimmune disease (please specify) -

* 3. Who confirmed your autoimmune or rheumatic disease diagnosis? (please tick all that apply)

- [ ] I am not sure
- [ ] Rheumatologist
- [ ] Neurologist
- [ ] Dermatologist
- [ ] Hematologist
- [ ] Endocrinologist
- [ ] Gastroenterologist
- [ ] Nephrologist/Kidney specialist/Renal Medicine
- [ ] Physician/Medicine doctor (internal medicine/ Internist)
- [ ] Primary care physician (PC), or family doctor or general practitioner (GP)
- [ ] Other doctor (please specify)

* 4. How old were you when you were first diagnosed with an autoimmune or rheumatic disease? (If you have both, please specify based on the rheumatic disease diagnosis)

1 120

* 5. Which type of myositis do you have? (please tick all that apply)

- [ ] I am not sure which type of myositis I have.
- [ ] Cancer-associated myositis
- [ ] Dermatomyositis (DM)
- [ ] Inclusion body myositis (IBM)
- [ ] Juvenile dermatomyositis (JDM)
- [ ] Necrotizing autoimmune myositis (NAM)
- [ ] Polymyositis (PM)
- [ ] I do not have myositis
- [ ] Other (please specify)

* 6. If you have ‘Overlap myositis’, which disease do you have in addition to myositis? (please tick all that apply)

- [ ] I am not sure
- [ ] Mixed connective tissue disease (MCTD)
- [ ] Rheumatoid arthritis
- [ ] Sjögren's syndrome
- [ ] Systemic lupus erythematosus/ lupus (SLE)
- [ ] Systemic sclerosis/ Scleroderma
- [ ] I do not have overlap myositis
- [ ] Other (please specify)

* 7. Are you aware which myositis antibodies you have? (please tick all that apply)?

- [ ] I am not sure if I have any antibodies
- [ ] I have antibodies, but I am not sure which one
- [ ] Anti-cN-1A (Mup44, NT5c1A)
- [ ] Anti-EJ
- [ ] Anti-HMGCR
- [ ] Anti-Jo-1
- [ ] Anti-KS
- [ ] Anti-Ku
- [ ] Anti-MDA-5/CADM-140
- [ ] Anti-Mi-2
- [ ] Anti-MJ/NXP-2
- [ ] Anti-PL-7
- [ ] Anti-PL-12
- [ ] Anti-PM/-Scl
- [ ] Anti-SAE
- [ ] Anti-SRP
- [ ] Anti-TIF1γ
- [ ] Anti-U1RNP
- [ ] Anti-U3RNP
- [ ] Other (please specify)

* 8. Has your doctor told you if you have antibodies specific for a connective tissue disease (like Lupus, Rheumatoid arthritis, scleroderma, Sjogren’s syndrome, etc)? If yes, which ones do you have? (please tick all that apply)

- [ ] I am not sure if I have any antibodies
- [ ] I have antibodies, but I am not sure which one
- [ ] Anti-Centromere antibody
- [ ] Anti- RNA Polymerase III
- [ ] Anti-Scl-70 (Anti-Topoisomerase-1 Antibody)
- [ ] Anti-Sm (Anti-smith antigen)
- [ ] Anti-Sm/RNP (Anti-Smith antigen and ribonucleoproteins)
- [ ] Anti-SSA/Ro
- [ ] Anti-SSB/La
- [ ] Anti-U1RNP antibody
- [ ] Antinuclear antibody (ANA)
- [ ] Anti-cyclic citrullinated peptides (CCP)
- [ ] Low complement (C3/C4)
- [ ] Rheumatoid Factor (RF)
- [ ] Anti-neutrophilic cytoplasmic antibody (ANCA)
- [ ] Lupus anticoagulant
- [ ] Anti-dsDNA antibody
- [ ] Other (please specify)

* 9. Has your SLE/lupus lead to kidney involvement/ lupus nephritis?

- ○ I am not sure
- ○ No
- ○ Yes

* 10. Do you have tophi/lumps of uric acid/urate/gout lumps anywhere on your body?

- ○ I am not sure
- ○ No, I don’t have any lumps related to gout on my body
- ○ Yes (ankle, foot, hand, wrist, forearm, elbow, knee, leg, earlobe)

* 11. How many gout attacks have you had over the last 6 months?

- ○ None
- ○ One
- ○ Two
- ○ Three
- ○ Four
- ○ Five or more

* 12. If you have Systemic Sclerosis/Scleroderma, which type of Scleroderma do you have?

- I am not sure
- I do not have scleroderma/systemic sclerosis
- My scleroderma INVOLVES skin thickening/tightening of my upper arms, thighs, chest, and/or abdomen/belly
- My scleroderma DOES NOT INVOLVE skin thickening/tightening of my upper arms, thighs, chest, and/or abdomen/belly
- My scleroderma DOES NOT INVOLVE the skin

* 13. If you have vasculitis, which type of vasculitis do you have?

- I do not have vasculitis
- I am not sure which type of vasculitis I have
- Eosinophilic granulomatosis with polyangiitis (Churg Strauss Syndrome)
- Giant cell arteritis
- IgA vasculitis (Henoch-Schönlein purpura)
- Kawasaki disease
- Microscopic polyangiitis
- Polyarteritis nodosa
- Takayasu’s arteritis
- Wegener's granulomatosis (Granulomatosis with polyangiitis)
- Other (please specify)

* 14. Have you been diagnosed with Interstitial lung disease (i.e., fibrotic/scarring lung disease), through a chest x-ray or CT scan in relation to your autoimmune or rheumatic condition?

- I am not sure
- No
- Yes

* 15. Have you been diagnosed with pulmonary arterial hypertension (PAH) related to your autoimmune/rheumatic condition?

- I am not sure
- No
- Yes

* 16. How old were you when first diagnosed with myasthenia gravis?

0 120

* 17. What type of myasthenia gravis do you have?

- ◯ Ocular
- ◯ Generalized
- ◯ I am not sure

If yes, for how many months have you had ocular/generalized myasthenia gravis?

* 18. Which tests were positive when you were diagnosed with myasthenia gravis?

- ☐ Repetitive nerve stimulation test (RNST)
- ☐ Single fiber electromyography (EMG)
- ☐ Edrophonium or Neostigmine test
- ☐ Icepack test
- ☐ Autoantibodies positivity
- ☐ I am not sure

* 19. What antibodies have you tested positive for?

- ☐ Anti- Ach R antibodies
- ☐ Anti-MuSK antibodies
- ☐ Anti LRP4 antibodies
- ☐ Cell-based assay
- ☐ I am negative for antibodies
- ☐ I am not sure
- ☐ Other (please specify)

* 20. Have you ever been diagnosed with thymoma?

- ◯ No
- ◯ Yes
- ◯ I am not sure

If yes, how many months ago?

* 21. Are you currently cured/in remission from thymoma?

- Yes
- No
- I am not sure

* 22. Have you experienced any myasthenic crises (breathing/swallowing difficulties requiring hospitalization)?

- No
- Yes

If yes, how many months ago?

* 23. What additional treatment did you receive during crises?

- [ ] Intravenous immunoglobulin (IVIG)
- [ ] Plasma exchange
- [ ] Intravenous Methyl Prednisolone/ oral high-dose steroids
- [ ] I am not aware.
- [ ] Other (please specify)

* 24. What medications are you currently taking for myasthenia gravis?

- [ ] Pyridostigmine
- [ ] Prednisone or prednisolone
- [ ] Azathioprine
- [ ] Mycophenolate Mofetil
- [ ] Methotrexate
- [ ] Cyclosporine
- [ ] Tacrolimus
- [ ] Intravenous immunoglobulin (IVIg) or Subcutaneous immunoglobulin (SCIg)
- [ ] Chronic plasma exchange
- [ ] Rituximab
- [ ] Complement inhibitor
- [ ] Neonatal Rc receptor (FcRn) blocker

For the next eight questions, please select your response that best describes your symptoms in the last 8 days to the Myasthenia Gravis -Activities of Daily Living scale

* 25. Talking

- Normal
- Intermittent slurring of nasal speech
- Constant slurring or nasal, but can be understood
- Difficult to understand speech

* 26. Chewing

- Normal
- Fatigue with solid food
- Fatigue with soft food
- Gastric tube

* 27. Swallowing

- Normal
- Rare episode of choking
- Frequent choking necessitating changes in diet
- Gastric tube

* 28. Breathing

- Normal
- Shortness of breath with exertion
- Shortness of breath at rest
- Ventilator dependence

* 29. Impairment of ability to brush teeth or comb hair

- None
- Extra effort, but no rest periods needed
- Rest periods needed
- Cannot do one of these functions

* 30. Impairment of ability to arise from a chair

- ○ None
- ○ Mild, sometimes uses arms
- ○ Moderate, always uses arms
- ○ Severe, request assistance

* 31. Double vision

- ○ None
- ○ Occurs, but not daily
- ○ Daily, but not constant
- ○ Constant

* 32. Eyelid droop

- ○ None
- ○ Occurs, but not daily
- ○ Daily, but not constant
- ○ Constant

Comorbidities

* 33. Has a doctor/physician ever diagnosed you with any of the following conditions? (please tick all that apply)

- [ ] No, I haven’t been diagnosed with any of these diseases
- [ ] AIDS/HIV infection
- [ ] Angina (chest pain)
- [ ] Asthma
- [ ] Chronic obstructive pulmonary disease (COPD), Acquired Respiratory Distress Syndrome (ARDS), or Emphysema (Chronic lung disease)
- [ ] Complicated diabetes (type I or II), i.e. I have one or more of the following health conditions as a result of my diabetes: coronary artery disease, stroke, peripheral arterial disease, retinopathy, nephropathy, or neuropathy.
- [ ] Congestive heart failure (or heart disease)
- [ ] Degenerative disc disease (back disease, spinal stenosis or severe chronic back pain)
- [ ] Dementia
- [ ] Hearing Impairment (very hard of hearing, even with hearing aids)- fill this only if you are experiencing any hearing loss
- [ ] Heart attack (myocardial infarct)
- [ ] Hemiplegia or paraplegia (Loss of control or weakness of half side of body or both legs)
- [ ] Hypertension
- [ ] Infertility
- [ ] Liver disease
- [ ] Neurological disease (such as multiple sclerosis or Parkinson’s disease)
- [ ] Obesity and/or body mass index >30 (weight in kg/height in meters^2)
- [ ] Osteoarthritis
- [ ] Osteoporosis
- [ ] Peptic ulcer disease
- [ ] Peripheral vascular disease
- [ ] Renal failure/ chronic kidney disease
- [ ] Stroke or TIA/transient ischaemic event (Cerebrovascular disease)
- [ ] Uncomplicated diabetes (type I or II)
- [ ] Upper gastrointestinal disease (ulcer, hernia, reflux).
- [ ] Visual impairment (such as cataracts, glaucoma, macular degeneration)
- [ ] Other (please specify)

____

* 34. Additionally, has a doctor/physician ever diagnosed you with any of the following health conditions? (please tick all that apply)

- [ ] No, I haven't been diagnosed with the given health conditions
- [ ] Fibromyalgia
- [ ] Chronic fatigue syndrome (CFS)
- [ ] Post-Covid syndrome
- [ ] Attention-deficit/hyperactivity disorder (ADHD)
- [ ] Autism
- [ ] Anxiety or panic disorder
- [ ] Bipolar disorder
- [ ] Dissociative disorder
- [ ] Depression
- [ ] Eating disorder
- [ ] Insomnia
- [ ] Obsessive-Compulsive disorder
- [ ] Phobia
- [ ] Post-traumatic stress disorder
- [ ] Schizophrenia
- [ ] Substance use disorder
- [ ] Any other health condition(please specify)

* 35. Have you ever been diagnosed with cancer? (please tick all that apply)

- [ ] I have never been diagnosed with cancer
- [ ] I am not sure/it is being investigated by the doctor
- [ ] Blood cancer (e.g., leukaemia, lymphoma, multiple myeloma)
- [ ] Breast
- [ ] Cervix
- [ ] Colon
- [ ] Head and neck cancer
- [ ] Lung
- [ ] Ovaries
- [ ] Prostate
- [ ] Skin and/or Melanoma
- [ ] Stomach
- [ ] Testicular
- [ ] Uterus
- [ ] Other (please specify)

* 36. When were you diagnosed with cancer?

- Before my diagnosis of autoimmune or rheumatic disease
- After my diagnosis of autoimmune or rheumatic disease
- At the same time as my diagnosis of autoimmune or rheumatic disease

* 37. At what age were you first diagnosed with cancer?

18 120

* 38. What is your smoking status?

- Non-smoker
- Ex-smoker
- Light (Less than 10 cigarettes per day)
- Moderate (10-19 cigarettes per day)
- Heavy (20 or over 20 cigarettes per day)

* 39. How often do you have a drink containing alcohol?

- I do not consume alcohol
- Once a month or less
- 2-4 times a month
- 2-3 times a week
- 4 or more times a week

Subjective well-being

* 40. Below are five statements that you may agree or disagree with. Please indicate your agreement with each item by choosing one of the options. Please be open and honest in your responding.

	Strongly disagree	Disagree	Slightly disagree	Neither agree nor disagree	Slightly agree	Agree	Strongly agree
In most ways, my life is close to my ideal.	◯	◯	◯	◯	◯	◯	◯
The conditions of my life are excellent.	◯	◯	◯	◯	◯	◯	◯
I am satisfied with my life.	◯	◯	◯	◯	◯	◯	◯
So far I have gotten the important things I want in life.	◯	◯	◯	◯	◯	◯	◯
If I could live my life over, I would change almost nothing.	◯	◯	◯	◯	◯	◯	◯

Current Health Status and Quality of Life

* 41. What is your current health status?

	Poor	Fair	Good	Very good	Excellent
In general, would you say your health is	◯	◯	◯	◯	◯
In general, would you say your quality of life is	◯	◯	◯	◯	◯
In general, how would you rate your physical health?	◯	◯	◯	◯	◯
In general, how would you rate your mental health, including your mood and your ability to think?	◯	◯	◯	◯	◯
In general, how would you rate your satisfaction with your social activities and relationships?	◯	◯	◯	◯	◯
In general, please rate how well you carry out your usual social activities and roles. (This includes activities at home, at work, and in your community, and responsibilities as a parent, child, spouse, employee, friend, etc.)	◯	◯	◯	◯	◯

* 42. To what extent are you able to carry out your everyday physical activities such as walking, climbing stairs, carrying groceries, or moving a chair?

◯ Not at all ◯ A little ◯ Moderately ◯ Mostly ◯ Completely

* 43. In the past 6 months, how often have you been bothered by emotional problems such as feeling anxious, depressed, or irritable?

◯ Always ◯ Often ◯ Sometimes ◯ Rarely ◯ Never

* 44. Over the last 6 months, what number between 0-10 best reflects your typical level of fatigue, if 0 means 'not at all fatigued' and 10 means 'extremely fatigued'?

0- not at all fatigued 10- extremely fatigued

* 45. Over the last 6 months, what number between 0-10 best reflects your typical level of pain, if 0 means 'no pain' and 10 means 'worst possible pain'?

0- no pain — 10- worst imaginable pain

* 46. Over the last 6 months, what number between 0-10 best reflects your severity of dryness, if 0 means 'no dryness' and 10 means 'maximum imaginable dryness'?

0- No dryness — 10- Maximum imaginable dryness

* 47. Are you able to

	Unable to do	With much difficulty	With some difficulty	With a little difficulty	Without any difficulty
Go for a walk for at least 15 minutes?	○	○	○	○	○
Run errands and shop?	○	○	○	○	○
Go up and down stairs at a normal pace?	○	○	○	○	○
Do chores such as vacuuming or yard work	○	○	○	○	○

Collating the Voice of people living with Autoimmune Diseases (CoVAD 3) Surv

For the next 6 questions, we would like to know how confident you are with self-management of your disease. For each of the following questions, please choose the number that corresponds to your confidence that you can do the tasks regularly at the present time.

* 48. How confident do you feel that you can keep the fatigue caused by your disease from interfering with the things you want to do?

Not at all confident- 1 Totally confident- 10

* 49. How confident do you feel that you can keep the physical discomfort or pain of your disease from interfering with the things you want to do?

Not at all confident- 1 Totally confident- 10

* 50. How confident do you feel that you can keep the emotional distress caused by your disease from interfering with the things you want to do?

Not at all confident- 1 Totally confident- 10

* 51. How confident do you feel that you can keep any other symptoms or health problems you have from interfering with the things you want to do?

Not at all confident- 1 Totally confident- 10

* 52. How confident do you feel that you can do the different tasks and activities needed to manage your health condition so as to reduce your need to see a doctor?

Not at all confident- 1 Totally confident- 10

* 53. How confident do you feel that you can do things other than just taking medication to reduce how much your illness affects your everyday life?

Not at all confident- 1 Totally confident- 10

Current Diseases Activity Status

* 54. In your opinion, how has your disease been in the last 3 months?

- I am not sure
- My disease is inactive or in remission
- My disease is active but stable and manageable
- My disease is active and improving
- My disease is active and worsening

* 55. Which part of your body has active rheumatic disease at the moment (please tick all that apply)?

- I am not sure
- None
- Joints
- Skin
- Muscle
- Lung
- Kidney
- Heart
- Brain
- Nerves
- Other (please specify)

* 56. Considering all the ways that your autoimmune/rheumatic disease affects you, please rate the overall activity of your disease today by placing a mark on the line below.

No evidence of disease activity (0)

Extremely active or severe disease activity (10)

* 57. Another process affecting health is disease damage, which is the effect on your body from previous disease activity and treatments for your autoimmune/rheumatic disease. Disease damage lasts at least six months (and sometimes forever) and is difficult (and sometimes impossible) to improve. Considering all the ways that your autoimmune/rheumatic disease affects you, **please rate the overall amount of damage your autoimmune/rheumatic disease has caused to your body by placing a mark on the line below.**

0 = No evidence of damage

10 = Extreme disease damage

* 58. Do you have swollen joints at the moment? If yes, how many? (spongy swelling with pain in joints, do not count bony enlarged joints or damaged joints)

- I am not sure
- I don’t have swollen joints
- 1-2 swollen joints
- 3-5 swollen joints
- 6 or more swollen joints

* 59. Are you currently experiencing any rashes that might be connected to your underlying rheumatic condition? If yes, could you please describe the rash? (please tick all that apply)

- I do not have any rashes
- Red rash around the eyes
- Red rashes on the knuckles
- Rashes on the knees
- Rashes on the thighs and/or hips
- Red rash on the cheeks
- Red rash in the V area of the chest (the upper chest area just below the neck)
- Red rashes on the outside of arms and/or forearm
- Mechanic’s hands (rough thick scaly skin on the fingers)
- Other (please specify)

* 60. Have your tests shown any of the following in the last 3 months? (please tick all that apply)

- I am not sure
- I did not undergo any laboratory/radiological testing
- MRI/ ultrasound suggesting active arthritis
- MRI/ ultrasound suggesting muscle inflammation
- Elevated inflammatory markers in blood (high ESR or CRP)
- Elevated muscle enzymes in blood (e.g., high CK or creatine kinase level)
- My tests were normal
- Other finding (please specify)

* 61. Has your doctor increased the dose of a medicine you were already receiving or added any new medicine for your rheumatic condition in the last 3 months?

- ◯ I am not sure
- ◯ No
- ◯ Yes

We want you to indicate how much your psoriatic arthritis impacts your health. Please tell us how you have been feeling in the last 6 months

* 62. Please slide the marker to indicate the number that best describes the pain you felt due to your psoriatic arthritis during the last 6 months:

0- None | 10- Extreme

* 63. Please slide the marker to indicate the number that best describes the overall level of fatigue due to your psoriatic arthritis you have experienced during the last 6 months:

0- No fatigue | 10- Completely exhausted

* 64. Please slide the marker to indicate the number that best describes the skin problems including itching you felt due to your psoriatic arthritis during the last 6 months:

0- None | 10- Extreme

* 65. Please slide the marker to indicate the number that best describes the difficulties you had to participate fully in work and/or leisure activities due to your psoriatic arthritis during the last 6 months:

0- None | 10- Extreme

* 66. Please slide the marker to indicate the number that best describes the difficulty you had in doing daily physical activities due to your psoriatic arthritis during the last 6 months:

0- No difficulty | 10- Extreme difficulty

* 67. Please slide the marker to indicate the number that best describes the feeling of discomfort and annoyance with everyday tasks due to your psoriatic arthritis during the last 6 months:

0- None | 10- Extreme

* 68. Please slide the marker to indicate the number that best describes the sleep difficulties (i.e., resting at night) you felt due to your psoriatic arthritis during the last 6 months:

0- No difficulty 10- Extreme difficulty

* 69. Considering your psoriatic arthritis overall, how well did you cope (manage, deal, make do) with your psoriatic arthritis during the last 6 months?

0- Very well 10- Very poorly

* 70. Please slide the marker to indicate the number that best describes the level of anxiety, fear and uncertainty (for example about the future, treatments, fear of loneliness) due to your psoriatic arthritis you have experienced during the last 6 months:

0- None 10- Extreme

* 71. Considering your psoriatic arthritis overall, Please slide the marker to indicate the number that best describes the level of embarrassment and/or shame due to your appearance experienced during the last 6 months:

0- None 10- Extreme

* 72. Please slide the marker to indicate the number that best describes the difficulties you had to participate fully in social activities (including relationships with family and/or people very close to you) due to your psoriatic arthritis during the last week:

0- None 10- Extreme

* 73. Please slide the marker to indicate the number that best describes the level of depression due to your psoriatic arthritis you have experienced during the last 6 months:

0- None 10- Extreme

Please place a mark on each line below to indicate your answer to each question relating to the past 6 months

* 74. How would you describe the overall level of pain/swelling in joints other than neck, back, and hips you have had?

0- None 10- Very severe

* 75. How would you describe the overall level of discomfort you have had from any areas tender to touch or pressure?

0- None 10- Very severe

* 76. How would you describe the overall level of morning stiffness you have had from the time you wake up?

0- None 10- Very severe

* 77. How long does your morning stiffness last from the time you wake up?

- 0 hours
- 1/2 hour
- 1 hour
- 1 and 1/2 hours
- 2 hours

* 78. During the past three months, what was the highest level of your CRP (C-reactive protein, an indicator of inflammation due to various reasons like infections, tissue injury, or other health issues)

- I am not sure
- Less than 1
- 1 to 3
- 4 to 10
- More than 10

Medication and adherence

* 79. Are you on/have you been on any of the following medications in the last 6 months (please tick all that apply)?

- [ ] I am not sure
- [ ] No, I haven’t taken any of these
- [ ] Allopurinol or Febuxostat
- [ ] Apremilast
- [ ] Avacopan
- [ ] Azathioprine
- [ ] Cyclophosphamide (Cytoxan)
- [ ] Cyclosporine
- [ ] Hydroxychloroquine
- [ ] IV immunoglobulin (IVIg) or Subcutaneous immunoglobulin (SQIg)
- [ ] JAK inhibitors (Tofacitinib, Baricitinib, Upadacitinib, Filgotinib, Peficitinib, Ruxolitinib, Brepocitinib)
- [ ] Leflunomide
- [ ] Methotrexate
- [ ] Mycophenolate mofetil or Mycophenolic acid
- [ ] Nintedanib
- [ ] Oral Tacrolimus
- [ ] Sulfasalazine
- [ ] Telitacicept
- [ ] URAT1 inhibitor AR882
- [ ] Voclosporin
- [ ] Anti TNF agents - Infliximab, Adalimumab, Certolizumab, Golimumab, Etanercept
- [ ] B cell inhibitors- Belimumab (Benlysta), Obinutuzumab (Gazyva, Gazyvaro)
- [ ] Rituximab
- [ ] Abatacept
- [ ] IL1 inhibitors – Anakinra, Canakinumab
- [ ] IL-6 inhibitors – Tocilizumab, Sarilumab, Siltuximab
- [ ] IL 12/23 inhibitors – Ustekinumab, Guselkumab (Tremfya), Risankizumab (Skyrizi), Tildrakizumab
- [ ] IL 17 inhibitors – Secukinumab, Ixekizumab, Brodalumab, Bimekizumab (Bimzelx)
- [ ] Type I IFN receptor inhibitors – Anifrolumab (Saphnelo)
- [ ] Other biologics or immunosuppressant/immunomodulator medicines (please specify)

[ ]

* 80. Are you currently on or have been on any steroids/corticosteroids (Prednisone, Medrol, Prednisolone, Wysolone, Omnacortil, etc.) in the last 6 months? (please check all that apply)

- [ ] I don’t recall if I am on steroids.
- [ ] No, I have never been on steroids
- [ ] No, I do not take any steroids now, but I have been on steroids before.
- [ ] Yes, < 5 mg/day
- [ ] Yes, 5-10 mg/day
- [ ] Yes, 10-20 mg/day
- [ ] Yes, >20 mg/day
- [ ] I have received intramuscular (in the muscle) steroid injections in the last 3 months
- [ ] I have received intravenous (in the blood) steroid injections in the last 3 months

* 81. Are you taking any cholesterol-lowering drugs currently? (please check all that apply)

- [ ] No, I haven't been taking any cholesterol-lowering drugs
- [ ] Statins (Atorvastatin, Fluvastatin, Lovastatin, Rosuvastatin, Simvastatin etc.)
- [ ] Fibrates (Gemfibrozil, Fenofibrate, Clofibrate, Ciprofibrate)
- [ ] Ezetimibe
- [ ] PCSK9 inhibitors (Alirocumab and Evolocumab)
- [ ] Bile acid binding resins (Cholestyramine, Colestipol, Colesevelam)
- [ ] ACL inhibitors (Bempedoic acid)
- [ ] Other (please specify)

* 82. Which statin are you currently taking?

- I am not sure which statin I am taking
- Atorvastatin
- Fluvastatin
- Lovastatin
- Pitavastatin
- Pravastatin
- Rosuvastatin
- Simvastatin
- Other (please specify)

* 83. For how many years have you been taking statins?

0 years — 40 years

* 84. Have you experienced any adverse events to cholesterol-lowering drugs?

- I don't know
- No
- Yes, muscle pain
- Yes, muscle weakness
- Yes, tender muscles
- Yes, I was diagnosed with myositis related to statins
- Other adverse events(please specify)

* 85. Are you on any of the following medications? (please tick all that apply)

- [ ] Allopurinol
- [ ] Colchicine
- [ ] Febuxostat
- [ ] NSAIDs (e.g., Indomethacin, Naproxen, Ibuprofen, etc)
- [ ] Pegloticase/Uricase
- [ ] Other (please specify)

* 86. Over the past 6 months, how often did you miss taking one or more of your prescribed medications?

- ( ) Never
- ( ) Once or twice in 6 months
- ( ) About once a month
- ( ) About once every week
- ( ) More than once every week

Collating the Voice of people living with Autoimmune Diseases (CoVAD 3) Surv

Personal Questions

* 87. What is your age?

18 years 120 years

* 88. To which gender do you identify/ what is your current gender identity?

- Prefer not to say
- Male
- Female
- Non-binary/third gender
- Transgender
- Prefer to self-describe (please specify)

* 89. Which country do you live in?

* 90. What is your race/ethnicity?

- Prefer not to say
- Asian
- Caucasian/White
- African
- Arab
- Hispanic
- Mixed ethnicity
- Other ethnicity (please specify)

* 91. What is your Asian ethnicity or cultural background?

- East Asian (e.g., Chinese, Japanese, Korean)
- Southeast Asian (e.g., Vietnamese, Thai, Filipino)
- South Asian (e.g., Indian, Pakistani, Sri Lankan)
- Central Asian (e.g., Kazakh, Uzbek, Kyrgyz)
- Other Asian (please specify)

* 92. Do you think of yourself as:

- Prefer not to say
- Straight/Heterosexual
- Gay or Lesbian
- Bisexual
- Queer
- Asexual
- Prefer to self-describe (please specify)

Social determinants of health

* 93. What is the highest grade or level of education that you have completed?

- No school
- Prefer not to disclose
- Primary school
- Secondary school
- Undergraduate university degree
- Postgraduate university degree

* 94. How much do you trust the healthcare system? (for example, hospitals, pharmacies, and other organizations involved in healthcare)

- Not at all
- A little
- Some
- A lot

* 95. Are you covered by any kind of health insurance or health care plan, including employer-sponsored insurance, prepaid plans, or government plans? (If you have two types of insurance, please choose the highest level of cover you have)

- [ ] I have no current health insurance/health care plan
- [ ] Yes, private health insurance, paid for by yourself
- [ ] Yes, private health insurance, paid for by employer
- [ ] Yes, public health cover, subsidized by the government

* 96. Which of the following applies to your situation?

	Strongly Agree	Agree	Neutral	Disagree	Strongly Disagree
Your health insurer cares more about saving money than about getting you the treatment you need.	○	○	○	○	○
You feel like you need to double-check everything your health insurer does.	○	○	○	○	○
You believe your health insurer will pay for everything it is supposed to, even really expensive treatments.	○	○	○	○	○
If you have a question, you think your health insurer will give you a straight answer.	○	○	○	○	○
All in all, you have complete trust in your health insurer.	○	○	○	○	○

* 97. Including yourself, how many people live in your household?

1 person 30 people

* 98. Which of the following best describe your current situation?

	Hardly ever	Some of the time	Almost always
I am satisfied with the help I receive from my family when something is troubling me	○	○	○
I am satisfied with the way my family discusses items of common interest and shares problem solving with me	○	○	○
I find that my family accepts my wishes to take on new activities or make changes in my life	○	○	○
I am satisfied with the way my family expresses affection and responds to my feelings	○	○	○
I am satisfied with the amount of time my family and I spend together	○	○	○

* 99. Which one of these comes closest to your own feelings about your household’s income?

- Living comfortably on present income
- Getting by on present income
- Finding it difficult on present income
- Finding it very difficult on present income

* 100. What is your current marital status?

- Married
- Living as married or living with a partner/spouse
- Divorced
- Widowed
- Separated
- Single, never been married
- Prefer not to say

* 101. Do you have regular caring responsibilities for children (under the age of 18), partner, elderly or disabled relatives? (mark all that apply)

- [ ] No
- [ ] Yes, for children (single parent)
- [ ] Yes, for children (NOT as a single parent)
- [ ] Yes, for children (divorced/ non co-inhabitant parent)
- [ ] Yes, for partner
- [ ] Yes, for elderly
- [ ] Yes, for disabled relatives

* 102. Are you employed (working for pay) at the moment?

- No
- Yes

* 103. Which of the following best describes what you are doing AT THE MOMENT (please tick all that apply)

- [ ] Working for an employer full-time (more than 30 hours per week)
- [ ] Working for an employer part-time (1 hour per week or more)
- [ ] Self-employed full-time (more than 30 hours per week)
- [ ] Self-employed part-time (1 hour per week or more)
- [ ] Working full-time in the home (domestic duties)
- [ ] Unemployed but seeking work
- [ ] Student
- [ ] Semi-retired
- [ ] Retired
- [ ] Other (please specify)

* 104. How would you characterize your present occupation? Please select one/more of the following options that best describe it.

- [ ] None of these
- [ ] White collar (involving sedentary work)
- [ ] Blue collar (involving active or manual work)
- [ ] Other (please specify)

* 105. If you are not currently employed, at what age did you stop working?

18 100

* 106. Do you think your disease was the main reason you stopped working?

- ( ) I can't say for sure
- ( ) No
- ( ) Yes

* 107. Which of these statements hold true for you?

	Strongly disagree	Disagree	Undecided	Agree	Strongly agree
I feel fairly satisfied with my present job	○	○	○	○	○
Most days I am enthusiastic about my work	○	○	○	○	○
Each day at work seems like it will never end	○	○	○	○	○
I find real enjoyment in my work	○	○	○	○	○
I consider my job to be rather unpleasant	○	○	○	○	○

Mental Health

* 108. Which of these statements accurately describe your situation?

	Often	Some of the time	Hardly ever
How often do you feel that you lack companionship?	◯	◯	◯
How often do you feel left out?	◯	◯	◯
How often do you feel isolated from others?	◯	◯	◯

* 109. Which of the following hold true for you?

	Strongly disagree	Disagree	Neutral	Agree	Strongly agree
I tend to bounce back quickly after hard times.	◯	◯	◯	◯	◯
It does not take me long to recover from a stressful event.	◯	◯	◯	◯	◯
I usually come through difficult times with little trouble.	◯	◯	◯	◯	◯

* 110. Additionally, which of these statements apply to you?

	Strongly agree	Agree	Neutral	Disagree	Strongly disagree
I have a hard time making it through stressful events.	◯	◯	◯	◯	◯
It is hard for me to snap back when something bad happens.	◯	◯	◯	◯	◯
I tend to take a long time to get over setbacks in my life.	◯	◯	◯	◯	◯

Pregnancy, Lactation and Family planning

* 111. Are you pregnant or lactating/breastfeeding currently or delivered in the last one year? (please check all that apply)

- No, I’m neither pregnant nor lactating currently
- Yes, I am pregnant
- Yes, I am lactating/breastfeeding
- Yes, I delivered in the last one year but I am not breastfeeding
- Other (please specify)

112. Is your partner pregnant or lactating/breastfeeding currently or has delivered in the last one year? (please check all that apply)

- No, my partner is neither pregnant nor lactating currently
- Yes, my partner is pregnant
- Yes, my partner is lactating/breastfeeding
- Yes, my partner delivered in the last one year but is not breastfeeding

* 113. What was the date of your/your partner's LMP (last menstrual period)?

Date / Time

Date

DD/MM/YYYY

* 114. What is the date of delivery or the expected delivery date?

Date / Time

Date

DD/MM/YYYY

* 115. Were assisted reproductive techniques such as IVF used for achieving the current pregnancy?

- Prefer not to say
- No
- Yes

* 116. Was a diagnosis established with any of the following diseases during the pregnancy? (please tick all that apply)

- [ ] No, I have/had no complications
- [ ] Antepartum Haemorrhage (Excessive bleeding before childbirth)
- [ ] Postpartum Haemorrhage (Excessive bleeding after childbirth)
- [ ] Hyperemesis (Severe nausea and vomiting during pregnancy) requiring medications
- [ ] Oligohydramnios (Low amniotic fluid during pregnancy)
- [ ] Preeclampia (High blood pressure during pregnancy)
- [ ] Eclampsia (Seizures during pregnancy due to high blood pressure)
- [ ] Gestational hypertension (High blood pressure during pregnancy, without other complications)
- [ ] Gestational diabetes (High blood sugar during pregnancy)
- [ ] Intrauterine growth restriction (IUGR) (Inadequate development of baby in the womb)
- [ ] Placental insufficiency (the placenta isn't nourishing the baby properly in the womb)
- [ ] IUD (Intrauterine death - death of baby in the womb before week 20 of gestation)
- [ ] Stillbirth (Death of the baby in the womb beyond week 20 of gestation)
- [ ] PROM (Premature rupture of membranes) (the fluid-filled sac surrounding the baby breaks before labor starts)
- [ ] Prolonged labour
- [ ] Infection
- [ ] Other (please specify)

* 117. Did your child have any complications after birth? (Please tick all that apply)

- [ ] No, my child didn’t/doesn’t have any complications
- [ ] LBW (Low Birth Weight)/Small for gestational age (SGA)
- [ ] Perinatal mortality (death of a baby either before birth (stillbirth) or within the first week of life)
- [ ] Infection requiring hospitalization
- [ ] Congenital malformations / Birth defects
- [ ] Other (please specify)

* 118. How many biological children do you have?

* 119. How many biological children did you initially plan to have?

Sexual Health and Contraception

* 120. Would you like to answer a few questions about your sexual health and contraception?

- ◯ No
- ◯ Yes

* 121. Which of the following are applicable to you? (please tick all that apply)

- [ ] I am not sexually active
- [ ] Prefer not to say
- [ ] I don’t use contraception
- [ ] I use/my partner uses barrier method of contraception (condoms, diaphragms, etc)
- [ ] I use/my partner uses hormonal contraception (birth control, intra-uterine device/IUD, etc)
- [ ] I use/my partner uses natural method of contraception (tracking menstrual cycle, withdrawal/pulling out, etc)
- [ ] I use/my partner have undergone tubal ligation/vasectomy (sterilization)
- [ ] I do not/my partner does not believe in using contraception
- [ ] I am/my partner is afraid of adverse events related to contraceptive methods
- [ ] I developed some issues after using contraception (please specify)

* 122. Indicate to what extent you agree with each statement below:

	I completely disagree	I disagree	I somewhat disagree	I neither agree nor disagree	I somewhat agree	I agree	I completely agree
The frequency of my sexual relations is satisfactory for me	○	○	○	○	○	○	○
There is nothing disturbing in my sex life	○	○	○	○	○	○	○
There’s a lot of physical pleasure in my sex life	○	○	○	○	○	○	○
I consider myself sexually fulfilled	○	○	○	○	○	○	○
I have no trouble realizing my sexual fantasies	○	○	○	○	○	○	○

Diet

* 123. Lastly, we would like to explore the impact that diet can have on your disease and quality of life.
Would you like to take a moment to tell us about your diet and food habits over the last 6 months?

- ◯ No
- ◯ Yes

* 124. Do you use olive oil as your main culinary fat?

- No
- Yes

* 125. How many tablespoons of olive oil do you consume per day? (Including olive oil used for cooking, salads, out of house meals, etc)

- No, I don't consume olive oil
- One or less
- Two or three
- Four or more

* 126. How many vegetable servings do you consume per day? (Including cooked and raw vegetables; potatoes and beans are not included). One serving = one large cup or half a large plate.

- No, I do not consume vegetables
- Less than one
- One
- Two
- Three or more

* 127. How many servings of fresh fruit do you consume per day?
One serving = one fruit unit of medium size, one large cup of sliced fruit, one slice of melon or watermelon of medium size, or one cup of freshly squeezed juice.

- No, I do not consume fresh fruit
- Less than one
- One
- Two
- Three or more

* 128. How many servings of red meat (pork, veal/beef, lamb), or red meat products (hamburgers, sausages, ham) do you consume per week? One serving = 100 to 150 g = a quarter to half a meal dish.

- No, I do not consume red meat/red meat products
- One or less
- Two to four
- Five to six
- Seven or more

* 129. How many servings of butter, margarine, or cream do you consume per day? One serving = 12 g = one dessert spoon for butter and margarine; 2 tablespoons for cream

- No, I do not consume butter, margarine, or cream
- Less than one
- One
- More than one

* 130. How many sweet beverages do you consume per day?

- No, I do not consume sweet beverages
- Less than one
- One
- More than one

* 131. How many glasses of wine do you consume per week?

- No, I do not consume wine
- One or less (occasionally)
- Two to six (sometimes but not daily)
- Seven to fourteen (one or two glasses per day)
- More than fourteen (more than two glasses per day)

* 132. How many servings of legumes do you consume per week? (beans, peas, chickpeas, lentils) One serving = 150 g = 3 table spoons

- No, I do not consume legumes
- Less than one
- One or two
- Three or more

* 133. How many servings of fish or shellfish do you consume per week? One serving = 100 to 150 g = a quarter to half a meal dish

- No, I do not consume fish or shellfish
- Less than one
- One
- Two
- Three or more

* 134. How many times per week do you consume commercial sweets or pastries (not homemade), such as cakes, cookies, biscuits, or custard?

- ◯ No, I do not consume commercial sweets or pastries
- ◯ Less than one
- ◯ One
- ◯ Two
- ◯ Three
- ◯ Four or more

* 135. How many servings of nuts do you consume per week? (nuts include non-salty peanuts, almonds, hazelnuts, chestnut, walnuts) One serving = 30 g = one handful

- ◯ No, I do not consume nuts
- ◯ Less than one
- ◯ One
- ◯ Two
- ◯ Three or more

* 136. Do you preferentially consume chicken, turkey or rabbit meat, or a vegetarian protein source, instead of red meat (veal/beef, pork, lamb) or any derived products (hamburgers, sausages, ham)?

- ◯ No
- ◯ Yes

* 137. How many times per week do you consume dishes cooked with tomato or tomato sauce, onion and (or) garlic, and olive oil?

- ◯ No, I do not consume dishes cooked with tomato or tomato sauce, onion and (or) garlic, and olive oil
- ◯ Less than one
- ◯ One
- ◯ Two or more

Exercise, Physical Activity & Digital Health

* 138. Which of the following types of exercises do you engage in regularly (by 'regularly', we mean at least 15 minutes every week)? (select all that apply)

- [ ] I do not exercise
- [ ] **Aerobic exercise** (Exercise intended to improve cardiorespiratory fitness and muscular endurance. Examples include walking, biking or cycling, running, hiking, aerobics, rowing, swimming, using an elliptical machine.)
- [ ] **Aquatic exercise** (Exercise performed in water, containing elements of both aerobic and resistance exercise. Examples include swimming, water aerobics, water walking or jogging.)
- [ ] **Resistance exercise** (Exercise intended to increase muscular strength. Examples include free weights, weight machines, resistance bands, pilates).
- [ ] **Mind–body exercise** (Exercise that combines movement, mental focus, and controlled breathing. Examples include yoga, Tai Chi, Qigong)
- [ ] Other exercises (please specify)

* 139. In the past 6 months, how often have you used an electronic wearable device (for example- Fitbit, fitness tracker watches, fitness tracking apps, etc.) to monitor or track your health or activity?

- ( ) I have not used a wearable device in the last 12 months
- ( ) Everyday
- ( ) Almost everyday
- ( ) 1-2 times per week
- ( ) Less than once per week

* 140. In the past 6 months, have you used the Internet to take care of any of the following health-related needs? (Please tick all that apply)

- [ ] I have not used the internet for these needs
- [ ] Look for health or medical information
- [ ] Send a message to a health care provider or a health care provider’s office.
- [ ] View medical test results
- [ ] Make an appointment with a healthcare provider

* 141. How did you learn about this survey?

- My physician
- My patient support group
- Social media

Other (please specify) or mention the name of your physician/ patient support group

142. If you are happy to share your email address below, we can contact you for follow-up regarding the progress of this survey:

Thank you for your time.
